# Virion-incorporated CD14 enables HIV-1 to bind LPS and initiate TLR4 signaling in immune cells

**DOI:** 10.1128/jvi.00363-24

**Published:** 2024-04-25

**Authors:** Arvin T. Persaud, Jasmin Khela, Claire Fernandes, Deepa Chaphekar, Jonathan Burnie, Vera A. Tang, Che C. Colpitts, Christina Guzzo

**Affiliations:** 1 Department of Biological Sciences, University of Toronto Scarborough, Toronto, Ontario, Canada; 2 Department of Cell and Systems Biology, University of Toronto, Toronto, Ontario, Canada; 3 Flow Cytometry and Virometry Core Facility, Department of Biochemistry, Microbiology, and Immunology, Faculty of Medicine, University of Ottawa, Ottawa, Canada; 4 Department of Biomedical and Molecular Sciences, Queen’s University, Kingston, Ontario, Canada; 5 Department of Immunology, Temerty Faculty of Medicine, University of Toronto, Toronto, Ontario, Canada; The Ohio State University, Columbus, Ohio, USA

**Keywords:** HIV-1, CD14, virion-incorporated proteins, innate immunity, TLR4 signaling, interferon regulatory factor, NF-κB, THP1-Dual cells, monocytes/macrophages, lipopolysaccharide (LPS), LPS receptor, inflammation, interferon, cytokines, flow virometry

## Abstract

HIV-1 has a broad range of nuanced interactions with the immune system, and the incorporation of cellular proteins by nascent virions continues to redefine our understanding of the virus-host relationship. Proteins located at the sites of viral egress can be selectively incorporated into the HIV-1 envelope, imparting new functions and phenotypes onto virions, and impacting viral spread and disease. Using virion capture assays and western blot, we show that HIV-1 can incorporate the myeloid antigen CD14 into its viral envelope. Virion-incorporated CD14 remained biologically active and able to bind its natural ligand, bacterial lipopolysaccharide (LPS), as demonstrated by flow virometry and immunoprecipitation assays. Using a Toll-like receptor 4 (TLR4) reporter cell line, we also demonstrated that virions with bound LPS can trigger TLR4 signaling to activate transcription factors that regulate inflammatory gene expression. Complementary assays with THP-1 monocytes demonstrated enhanced secretion of inflammatory cytokines like tumor necrosis factor alpha (TNF-α) and the C-C chemokine ligand 5 (CCL5), when exposed to LPS-loaded virus. These data highlight a new type of interplay between HIV-1 and the myeloid cell compartment, a previously well-established cellular contributor to HIV-1 pathogenesis and inflammation. Persistent gut inflammation is a hallmark of chronic HIV-1 infection, and contributing to this effect is the translocation of microbes across the gut epithelium. Our data herein provide proof of principle that virion-incorporated CD14 could be a novel mechanism through which HIV-1 can drive chronic inflammation, facilitated by HIV-1 particles binding bacterial LPS and initiating inflammatory signaling in TLR4-expressing cells.

**IMPORTANCE:**

HIV-1 establishes a lifelong infection accompanied by numerous immunological changes. Inflammation of the gut epithelia, exacerbated by the loss of mucosal T cells and cytokine dysregulation, persists during HIV-1 infection. Feeding back into this loop of inflammation is the translocation of intestinal microbes across the gut epithelia, resulting in the systemic dissemination of bacterial antigens, like lipopolysaccharide (LPS). Our group previously demonstrated that the LPS receptor, CD14, can be readily incorporated by HIV-1 particles, supporting previous clinical observations of viruses derived from patient plasma. We now show that CD14 can be incorporated by several primary HIV-1 isolates and that this virion-incorporated CD14 can remain functional, enabling HIV-1 to bind to LPS. This subsequently allowed CD14^+^ virions to transfer LPS to monocytic cells, eliciting pro-inflammatory signaling and cytokine secretion. We posit here that virion-incorporated CD14 is a potential contributor to the dysregulated immune responses present in the setting of HIV-1 infection.

## INTRODUCTION

The human immunodeficiency virus (HIV) is an enveloped retrovirus that acquires its lipid bilayer as it egresses through the plasma membrane of infected cells (1–4). As a result, host proteins (HPs) present at the sites of virus budding are incorporated into nascent virus particles, and impart an antigenic phenotype to the viral surface that is similar to the infected cell surface (5–10). Many virion-incorporated HPs remain biologically active on the viral surface, and can confer new biological functions onto virions, including altered virus homing and attachment to target cells (11–22). For example, the incorporation of integrin α4β7 enables HIV-1 virions to transit more efficiently to gut-associated lymphoid tissues (11), which are coincidentally home to a large population of HIV target cells, CD4^+^ T cells (23, 24). More recently, our work has also shown that virion-incorporation of CD162 (P-selectin glycoprotein ligand-1) can enable HIV-1 virions to be captured and transferred to susceptible cells by interaction with CD62P (P selectin), the cognate ligand of CD162 (25).

Beyond attachment, some incorporated HPs allow virions to participate in signal transduction events, thereby licensing them with immunomodulatory functions. Work from Tremblay et al. (26, 27) showed that HIV-1 can incorporate CD86 (B7-2) which, when coupled with CD3 ligation, can provide costimulatory signals to T cells. Most interesting was the resulting activation of the transcription factors nuclear factor kappa B (NF-κB) and nuclear factor of activated T cells, which activated HIV-1 long-terminal repeat-driven gene expression (27). More recently, it was shown that HIV-1 can incorporate the immune checkpoint ligand, programmed death-ligand 1 (PD-L1) (28). Conferred with an immunoregulatory ability, HIV-1 virions with incorporated PD-L1 impaired follicular helper T cell proliferation and cytokine secretion, resulting in reduced IgG1 secretion by autologous B cells.

In this study, we focus on the incorporation of the myeloid antigen, CD14, for its role in mediating inflammatory responses. Indeed, chronic immune activation and inflammation are hallmarks of HIV-1 pathogenesis, and are largely mediated by dysregulated cytokine production (29, 30). Lawn and colleagues were among the first to demonstrate that CD14 was a reliable marker for macrophage-derived HIV-1 from infected patients (31–33), but studies investigating the functional implications of virion-incorporated CD14 are still lacking. We sought to bridge this knowledge gap and leverage our flow virometry (FVM) techniques to describe the function of virion-incorporated CD14. Flow virometry (or nanoscale cytometry) methodology relies on flow cytometric principles but is performed using instrumentation optimized to detect nanoscale particles, like individual virions. Additionally, the use of fluorescence reference beads in compliance with standardized frameworks enables a quantitative estimate of antigen abundance on the surface of virions and reports these data in calibrated units (34, 35). Though nanoscale cytometry is extensively used in the extracellular vesicles (EVs) field, our lab has adapted these techniques for the study of cellular proteins incorporated into the HIV-1 envelope (25, 36, 37) and applied them here to investigate CD14 incorporation by HIV-1.

CD14 is a non-signaling, glycosylphosphatidylinositol (GPI)-anchored protein that belongs to a class of germline-encoded receptors, termed pattern recognition receptors, for their recognition of conserved microbial moieties. Specifically, CD14 recognizes and binds lipopolysaccharide (LPS) and, coupled with the signaling capacity of Toll-like receptor 4 (TLR4), this comprises an early step in innate immune responses against gram-negative bacteria (38–40). Interestingly, the envelope glycoprotein of HIV-1 (41) and other enveloped viruses (42) can also activate TLR4, though the role of CD14 remains undefined in those contexts. Both CD14 and TLR4 were previously shown to be exploited by a mouse retrovirus for establishing tolerance in infected mice (43), offering potential insights of what may happen in the context of HIV-1. Herein, we investigated the biological activity of CD14 on the surface of HIV particles, demonstrating for the first time that CD14^+^ virions are poised to bind bacterial LPS. This preservation of biological activity proved significant, as it facilitated the shuttling of LPS by HIV-1 virions to human monocytes, provoking inflammatory signaling and cytokine secretion.

## RESULTS

### CD14 is incorporated into primary HIV-1 isolates

**Fig 1:**
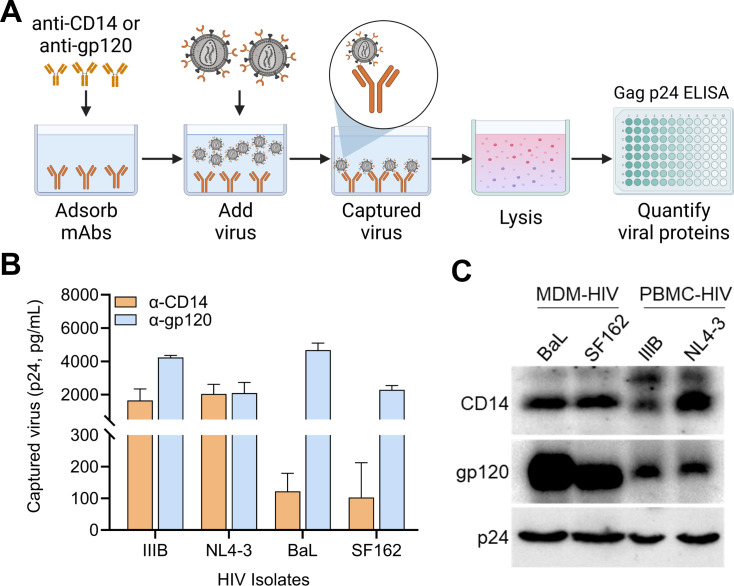
Screening for CD14 incorporation by lab-adapted HIV-1 isolates. (**A**) Schematic describing the virion capture assay used throughout. Briefly, antibodies or LPS (not depicted) were adsorbed onto 96-well plates and blocked before adding virus samples to allow precipitation of virus particles bearing the antigen of interest. Unbound virions were washed away and captured virions were lysed for quantitation of HIV-1 by Gag_p24_ ELISA. (**B**) Virion capture assay was performed on four HIV-1 isolates (IIIB, NL4-3, BaL, and SF162) produced by infected PBMCs using anti-CD14 (orange) and anti-gp120 (blue). Bars are isotype control-subtracted mean ± SD Gag_p24_ of duplicate assays and are representative of two independent experiments. (**C**) Western blot corroborating the incorporation of CD14. HIV-1 grown in monocyte-derived macrophages (MDMs) (BaL and SF162, two donors each) and PBMCs (IIIB, two donors; NL4-3, four donors) were concentrated, lysed, and resolved by SDS-PAGE before blotting for p24 (loading control), gp120 (positive control for viral surface protein), and CD14. Data are representative of three independent blots.

Virion capture assays (VCA, Fig. 1A) are routinely used by our group and others to characterize various virion-incorporated cellular proteins (5, 25, 36). As an initial test of the extent of CD14 incorporation into HIV virions, we screened a small panel of peripheral blood mononuclear cell (PBMC)-derived HIV-1 isolates (HIV-1_IIIB_, HIV-1_NL4-3_, HIV-1_BaL_, and HIV-1_SF162_) by VCA (Fig. 1A), testing the immunoprecipitation of virions with an anti-CD14 monoclonal antibody (mAb) (clone M5E2) (Fig. 1B, orange bars). As a positive control, virions were also immunoprecipitated by a mAb against the surface glycoprotein of HIV-1, anti-gp120 (clone PG9) (Fig. 1B, blue bars).

Next, as an orthogonal technique to corroborate our initial findings, we performed immunoblotting on polyethylene glycol (PEG)-concentrated virus, pooling matched isolates from multiple primary cell cultures to get a broader representation of virion-incorporated CD14 across diverse donors (Fig. 1C). Since CD14 is conventionally a marker for the myeloid lineage and macrophages are one of the main target cells for HIV infection, we also included HIV-1 isolates propagated in monocyte-derived macrophages (MDMs) in our immunoblotting. The HIV capsid protein, Gag_p24_, was blotted for as a loading control (Fig. 1C, bottom row, “p24”), in addition to gp120 as a positive control (middle), representing an antigen known to be incorporated into the viral envelope. CD14 was present in viral lysates from both MDMs and PBMC isolates tested (top row). These results support previous reports detecting HIV-incorporated CD14 in clinical samples from patients with bacterial or parasitic co-infections (31–33, 44). Moreover, it also supports widespread CD14 incorporation by HIV-1 isolates propagated in different primary cell cultures in the absence of co-infections.

### CD14 is efficiently incorporated into HIV-1 pseudovirus (PV) particles

**Fig 2:**
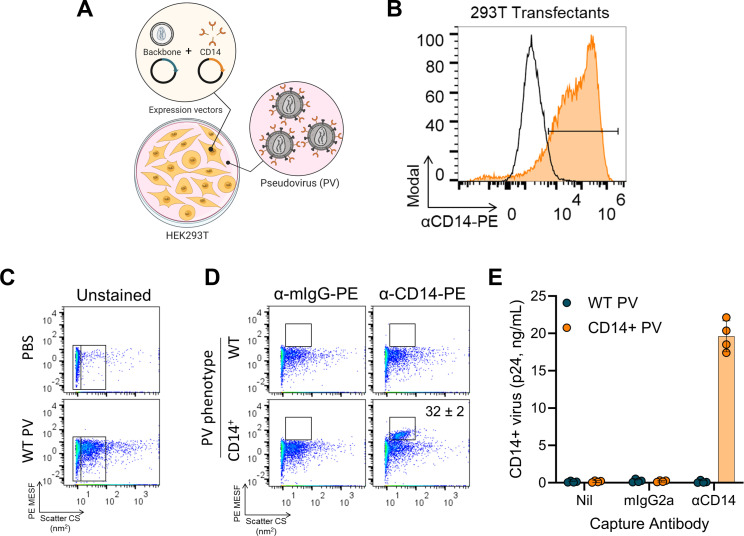
HIV-1 pseudovirus reliably models virion incorporation of CD14. (**A**) Schematic depicting the process of transfecting 293T cells with plasmids encoding the HIV-1 backbone (pSG3ΔEnv) and CD14 to produce PV bearing CD14 on the surface. (**B**) Confirming the transfection efficiency of 293T cells ectopically expressing CD14. Cells were transfected with backbone only (black histogram) or backbone plus CD14 (orange histogram) to produce WT or CD14^+^ PVs, respectively. Cells were collected and stained with PE-conjugated anti-CD14 to confirm cell-surface expression of CD14 available for incorporation. (**C**) Gating strategy to identify PV populations by FVM. Phosphate-buffered saline (PBS) or WT virions were acquired, and data are displayed on pseudocolored dot plots. Fluorescence data (y-axis) were calibrated to produce quantitative units of MESF (molecules of equivalent soluble fluorophore), which describe the number of fluorophores and, indirectly, the number of antigens detected. Side scatter data (x-axis) were calibrated to produce arbitrary units of scattering cross section (nm^2^), which relates to the size and refractive index of particles. Gates to the far left (PBS and WT PV) represent the instrument’s optical noise and are present of every FVM plot. Virions are identified as the dense, monodispersed population (right gate) adjacent to the optical noise. (**D**) WT (top row) and CD14^+^ (bottom row) virions were stained with PE-conjugated isotype control (left column) or anti-CD14 (right column) (0.4 µg/mL) and analyzed by FVM. Gates were positioned above the unstained PV populations, identifying PE^+^ events, and were set respective to each isotype control stain. PE MESF values are enumerated where PE^+^ events are present (mean ± SD, *n* = 3 independent stains), describing the number of fluorophores detected in each PE^+^ gate. Virus stocks used in C and D had a titer of 187 ng/mL (WT) and 114 ng/mL (CD14^+^) of p24 as measured by ELISA. (**E**) Virion capture assay was performed on WT (teal) and CD14^+^ (orange) PVs to corroborate CD14 incorporation. “Nil” represents PBS buffer, “mIgG2a” is an isotype-matched control, and “αCD14” is anti-CD14 mAb M5E2. Bars are mean ± SD p24 of four independently produced virus stocks and are representative of at least two independent assays.

We next wanted to determine whether virion-incorporated CD14 confers additional biological functions to HIV-1 virions. Our recent work highlights the usefulness of pseudoviral particles as models for functional studies on virion-incorporated proteins (25, 36, 37), and we therefore turned to PV models of HIV-1 for this study. These PVs are produced by co-transfection of 293T cells with plasmids encoding the HIV-1 backbone (pSG3ΔEnv) and cellular CD14, as depicted in Fig. 2A (36). Optimized production of PV particles is also outlined in Fig. S1, where we titrated the amount of CD14 in the viral envelope by transfecting increasing amounts of CD14 plasmid DNA (pDNA; Fig. S1A and B). We selected 0.25 µg as the optimized pDNA condition for CD14^+^ PV production, since higher amounts of pDNA negatively affected the health of the transfected cells and compromised the distribution of the virus population (Fig. S1A, “1 µg”). We also performed antibody titrations with the anti-CD14-PE (phycoerythrin) and isotype-matched control (Fig. S1C and D) antibodies to determine the optimal staining conditions of PVs with FVM. As expected, the anti-CD14-PE did not show any appreciable staining on wild-type (WT) PVs that are devoid of CD14 (Fig. S1C, bottom row), and the negligible staining observed was comparable to the isotype control staining (Fig. S1C, top row; S1D, hatched black bars). The level of specific staining on CD14^+^ virions correlated with the increasing anti-CD14 mAb concentrations (Fig. S1C, middle row and S1D, orange bars). Stain index (SI; Fig. S1D, magenta line) peaked at 0.8 µg/mL mAb and subsequently plateaued, indicating that a maximal signal is achieved at that concentration, and therefore, we selected a mAb concentration of 0.4 µg/mL for subsequent experiments.

Before performing experiments with the optimized PV particles, we first confirmed cellular expression of CD14 on the HIV-1 PV producer cells (Fig. 2B, orange vs black histograms). Next, we performed FVM to quantify the incorporation of CD14 on our HIV-1 PVs. To set our instrument parameters and gating strategies for detecting PV particles by light scatter, we first acquired unstained PBS (phosphate-buffered saline) controls (Fig. 2C, top) alongside unstained PVs (bottom), and we observed the PVs appearing as a distinct, monodispersed population (right gate), along with the optical noise of the instrument (left gate), as expected (25, 36, 37).

Next, we performed immunolabeling with PE-conjugated anti-CD14 or isotype-matched controls, on WT PVs and CD14^+^ PVs. The acquired FVM data were calibrated on FCM_PASS_ (45) using PE Quantibrite beads to report standardized fluorescence units in PE molecules of equivalent soluble fluorophores (PE MESF, enumerated in Fig. 2D). Given the large size of the PE fluorophore, conjugated antibodies typically exhibit a fluor:protein ratio of 1:1, which allows us to approximate the number of antigens labeled by first quantifying the number of fluorophores detected. Thus, 1 PE MESF corresponds to approximately one to two labeled antigens, given the bivalent nature of these mAbs. The gates defining PE positive staining was established using the isotype controls, which produced negligible PE^+^ events for both PV phenotypes (Fig. 2D, α-mIgG-PE). Since these samples had no reportable PE MESF values, there is no MESF annotation on the isotype-stained plots in Fig. 2D. In contrast, anti-CD14 staining on CD14^+^ PVs produced a mean PE MESF of 32 ± 2 SD (*n* = 3), indicating that PVs can contain ~32 molecules of CD14 antigen on their surface (Fig. 2D, α-CD14-PE), while the WT PV stained with α-CD14-PE did not show any reportable PE MESF.

An advantage of our FVM protocols is that we can assay virus particles directly from culture supernatants, without any additional processing that may bias surface proteins on viral particles. Given that we are assaying culture supernatants, the events displayed on the dot plots can include EVs, in addition to virus particles, since they are ubiquitously produced, and these EVs can overlap in size with viruses and contain similar antigenic profiles (46, 47). Therefore, although FVM data provide advantages in allowing us to look at virion heterogeneity and distribution, it is most appropriately corroborated with complementary techniques that can specifically identify virus particles. Thus, we performed virion capture assays on our PVs by precipitating virions with anti-CD14 antibodies (Fig. 2E), and quantified the captured virus by measuring Gag p24 as an HIV-1-associated structural protein. WT PVs (teal) were not precipitated regardless of capture conditions, whereas CD14^+^ PVs (orange) were precipitated in the anti-CD14 condition but not with PBS buffer (“Nil”) or isotype control mAb (“mIgG2a”), confirming that the CD14^+^ events gated in Fig. 2D were intact virions and unlikely to be EVs. While we cannot definitively exclude EVs from being precipitated in these capture assays, the readout of Gag p24 minimizes the contribution of EVs to these data, as others have shown very low levels of Gag p24 associated with EVs (48, 49).

### Virion-incorporated CD14 remains biologically active and can bind LPS

**Fig 3:**
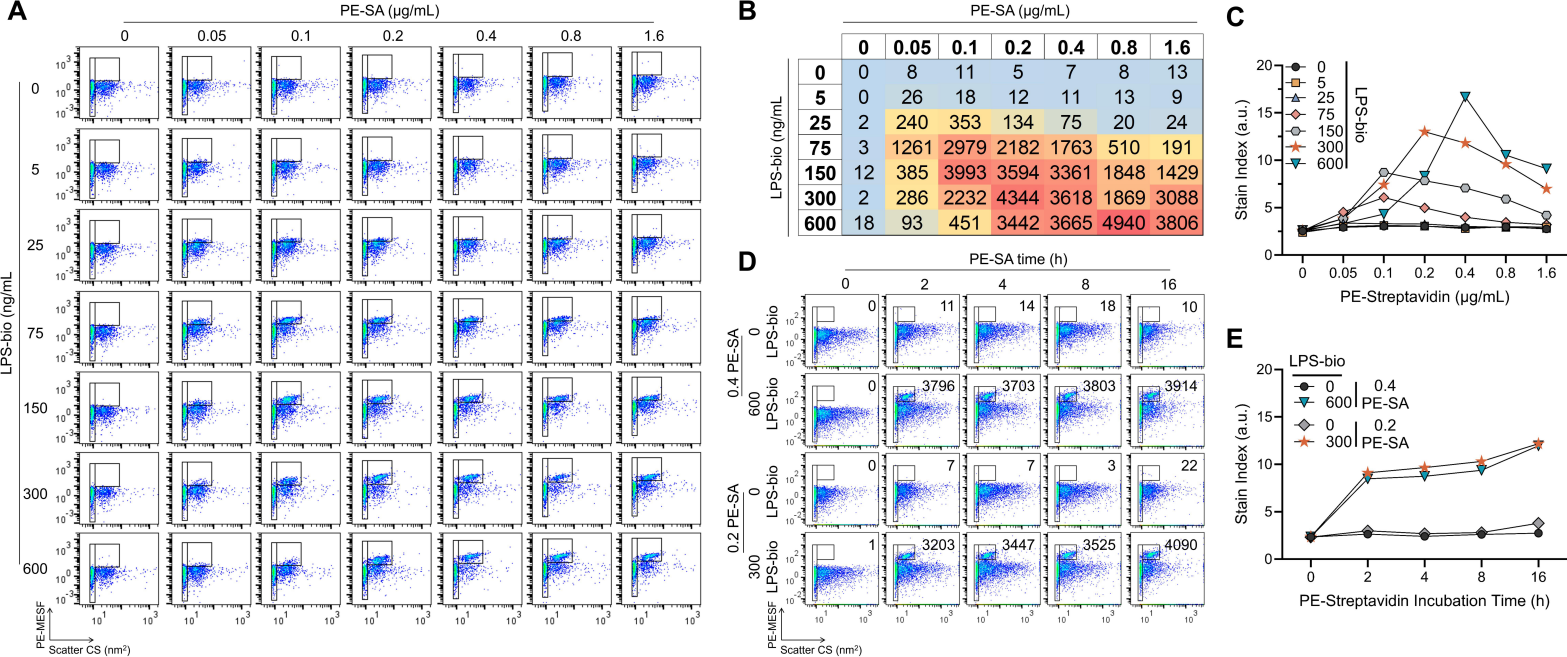
CD14 remains functional in the viral envelope and binds to LPS. (**A**) FVM was used to read out a checkboard titration of LPS-bio and PE-SA on CD14^+^ virions to identify optimal staining concentrations. LPS-bio was incubated overnight at 4°C with virus samples and PE-SA was added for 4 h at 4°C the next day before acquisition. Gates to the left identify optical noise used for (**C**), and gates to the right identify PE^+^ events that were set respective to each negative control PE-SA stain (top row). CD14^+^ virus stock shown in FVM plots had a titer of 57 ng/mL p24 as measured by ELISA. (**B**) The number of particles in the PE^+^ gates of (**A**) were tabulated and heat mapped with blue, yellow, and red identifying low, intermediate, and high values, respectively. Higher particle counts identify LPS-bio and PE-SA stains that support CD14^+^ virions binding. (**C**) SI was calculated and plotted for each PE-SA concentration using the PE MESF measured in the PE^+^ gates of (**A**). Peak SI identifies optimal staining pairs (orange stars and blue inverted triangles). (**D**) Incubation times for optimal PE-SA pairs from (**C**) were evaluated over a 16-h time course. Particle counts in PE^+^ gates (right gates) are enumerated on each plot. CD14^+^ virus stock shown in FVM plots had a titer of 73 ng/mL p24 as measured by ELISA. (**E**) SI of PE MESF measured in (**D**) were plotted over time. All titration data were reproducible across two independent experiments using two independently produced virus stocks. All LPS-bio and PE-SA concentrations in A–E are in units of ng/mL and µg/mL, respectively.

Next, we evaluated the biological activity of virion-incorporated CD14, by testing virion binding to bacterial LPS. We were interested but had difficulty in obtaining a commercial PE-conjugated LPS and opted for an Alexa Fluor 488-LPS conjugate instead (LPS-AF488). We first evaluated binding of LPS-AF488 to 293T cells ectopically expressing CD14 (Fig. S2A) before attempting to test binding to CD14^+^ virions (Fig. S2B). In our hands, we were unable to detect appreciable LPS-AF488 binding to either transfected cells or to virions with incorporated CD14, regardless of the amount of LPS tested (0 ng/mL–1,000 ng/mL). Binding to PVs was marginal at best (Fig. S2B), despite a generous gating strategy including the entire virion population to account for lower levels of LPS binding. In parallel, we also tested indirect staining methods with biotinylated LPS (LPS-bio) as the primary stain, and PE-streptavidin (PE-SA) secondary detection on transfected cells [Fig. S2A and CD14^+^ virions (Fig. S2C; Fig. 3)]. On cells, indirect staining with LPS-bio and PE-SA (Fig. S2A) yielded much stronger signals compared to direct staining with LPS-AF488 (Fig. S2A, middle column), and even reproduced the bimodal distribution of CD14-transfected cells revealed by mAb staining (Fig. S2A, left column). On pseudoviruses, we also observed superior LPS signals with indirect staining methods (LPS-bio and PE-SA), compared to direct staining with LPS-AF488 (Fig. S2C and D; Fig. 3). LPS-bio and PE-SA proved to be efficient and sensitive methods to detect LPS binding to virions with discernable staining as early as 5 min into incubation with PE-SA (Fig. S2C and D). Even after 4 h of PE-SA, SI calculations revealed an increasing trend in PE signal (Fig. S2D).

Given that our flow virometry staining protocols do not include wash steps, we performed a more thorough characterization of LPS-bio binding and PE-SA staining by titrating LPS-bio and PE-SA concentrations on CD14^+^ virions (Fig. 3A). Counts of PE^+^ particles (Fig. 3A, right gates) were tabulated and heat mapped (Fig. 3B), revealing the LPS-bio and PE-SA concentrations that support binding by CD14^+^ virions, evidenced by higher particle counts shaded in red. SI plots (Fig. 3C) identified two optimal pairs for staining (300 ng/mL LPS-bio + 0.2 µg/mL PE-SA, and 600 ng/mL LPS-bio + 0.4 µg/mL PE-SA) based on peak SI (orange stars and inverted blue triangles, respectively). SI for the former condition (300 + 0.2) was slightly higher over a 16-h PE-SA time course (Fig. 3E, orange stars), and since this condition allowed us to introduce less fluorophores and LPS during staining, 300 ng/mL LPS-bio + 0.2 µg/mL PE-SA was selected as the optimal pair (Fig. 3C and E, orange).

### Characterizing the specificity of HIV-incorporated CD14 for LPS binding

**Fig 4:**
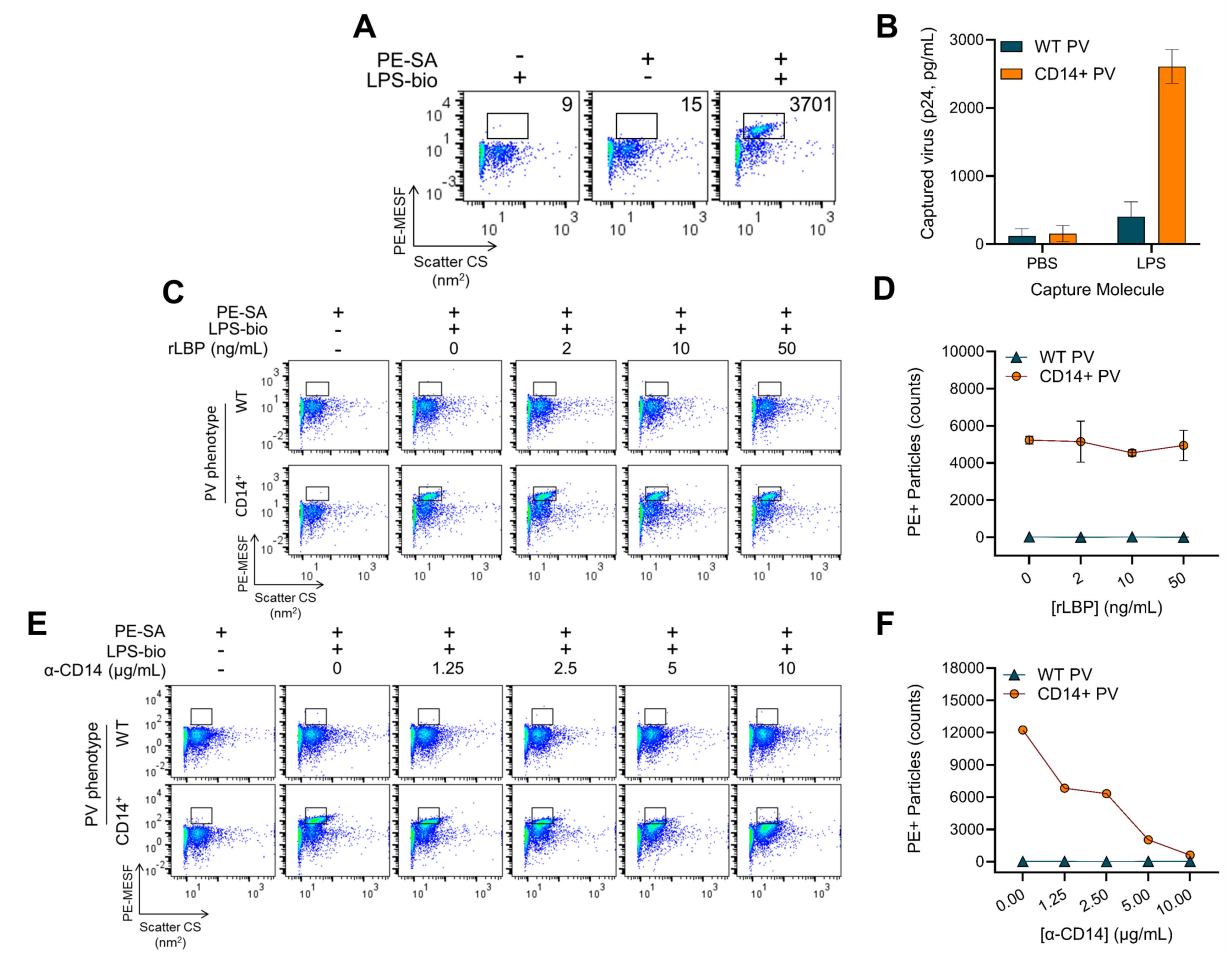
LPS binds to HIV-1 in a CD14-specific manner and does not passively associate with the lipid envelope. (**A**) Optimized LPS-bio (300 ng/mL, overnight, 4°C) and PE-SA (0.2 µg/mL, 4 h, 4°C) staining on CD14^+^ virions. Particle counts in PE^+^ gates are enumerated on each plot. (**B**) Virion capture assay using immobilized untagged LPS. Ninety-six-well plates were coated with buffer (PBS) or LPS and virions with (CD14^+^, orange bars) or without CD14 (WT, teal bars) were added. Captured virions were quantified by Gag_p24_ ELISA. Bars are mean ± SD of duplicate capture assays and are representative of three independent experiments. (**C**) FVM evaluating the contribution of recombinant LPS-binding protein (LBP) on LPS-bio binding to CD14^+^ virions. Varying amounts of recombinant LBP (rLBP) were mixed with LPS-bio prior to incubating with WT (top row) and CD14^+^ (bottom row) virus samples and staining with PE-SA. Gates define PE^+^ events based on negative control (PE-SA only). WT and CD14^+^ virus stocks shown in FVM plots for (**A**) and (**C**) had a respective titer of 76 and 57 ng/mL p24 as measured by ELISA. (**D**) Particle counts in PE^+^ gates from (**C**) were plotted as a function of PE-SA concentrations for WT (teal triangles) and CD14^+^ virions (orange circles). Data are mean ± SD of two independent virus stocks. (**E**) FVM measuring neutralization of LPS-bio binding to virions. Anti-CD14 (M5E2) was added to WT (top row) and CD14^+^ (bottom row) virions before incubation with LPS-bio and PE-SA. Gates are as described for (**C**). WT and CD14^+^ virus stocks shown in FVM plots had a respective titer of 368 and 141 ng/mL p24 as measured by ELISA. (**F**) Particle counts in PE^+^ gates from (**E**) were plotted over the range of anti-CD14 concentrations tested for WT (teal triangles) and CD14^+^ (orange circles) virions. Data are representative of two independent virus stocks.

Next, we applied our optimized indirect staining conditions, and observed reproducible and robust LPS labeling on CD14^+^ virions (Fig. 4A). However, one caveat of indirect staining techniques is the loss of protein quantitation since streptavidin contains four binding sites for biotin and the indirect staining protocol inherently saturates the PVs with PE-SA. Therefore, we corroborated the FVM detection of LPS binding to PVs with orthogonal techniques by performing VCA. We immobilized purified, unlabeled LPS on a 96-well plate (Fig. 4B) and observed high levels of captured CD14^+^ virions (orange bars), while no LPS-mediated capture of WT PVs (teal bars) and no capture on PBS-coated wells were observed for either PVs.

To further probe biological activity, we tested whether LPS-binding protein (LBP), a soluble protein that presents LPS to CD14 and TLR4, can increase the amount of LPS bound to CD14^+^ virions. We incubated LPS-bio with increasing amounts of recombinant LBP (rLBP; Fig. 4C) before adding to WT PVs (top row) or CD14^+^ PVs (bottom row). No significant enhancement or antagonistic effect was observed with rLBP, as demonstrated by the stable particle counts across the tested concentrations (staining plots in Fig. 4C and enumerated in Fig. 4D). This is consistent with the results of flow cytometry on cells with LPS-bio and PE-SA, where rLBP had no impact on staining (Fig. S2A).

Structurally, LPS is a heavily branched sugar linked to a lipid A tail. Through hydrophobic lipid-lipid interactions, it is possible that LPS can passively be inserted into the phospholipid bilayer of the HIV-1 envelope, as recently demonstrated for extracellular vesicles (50). Though we observed negligible binding of LPS-bio to WT virions (Fig. 4C and E; top rows), we sought to definitively confirm the specificity of the CD14-LPS interaction on CD14^+^ HIV-1. To that end, we added increasing concentrations of anti-CD14 (mAb M5E2) prior to loading virions with LPS-bio and detecting with PE-SA (Fig. 4E and F). Supporting the specific dependence on CD14, LPS-bio binding to CD14^+^ HIV-1 was effectively blocked in a concentration-dependent manner by anti-CD14. This was evidenced by the reduction of PE^+^ particles toward control levels (Fig. 4F, orange circles) with increasing amounts of anti-CD14. Therefore, we expect that passive lipid-lipid interactions do not play a significant role in LPS binding to the pseudovirions tested in this study.

### CD14^+^ HIV-1 can deliver bioactive LPS to human monocytes for inflammatory signaling through NF-κB

**Fig 5:**
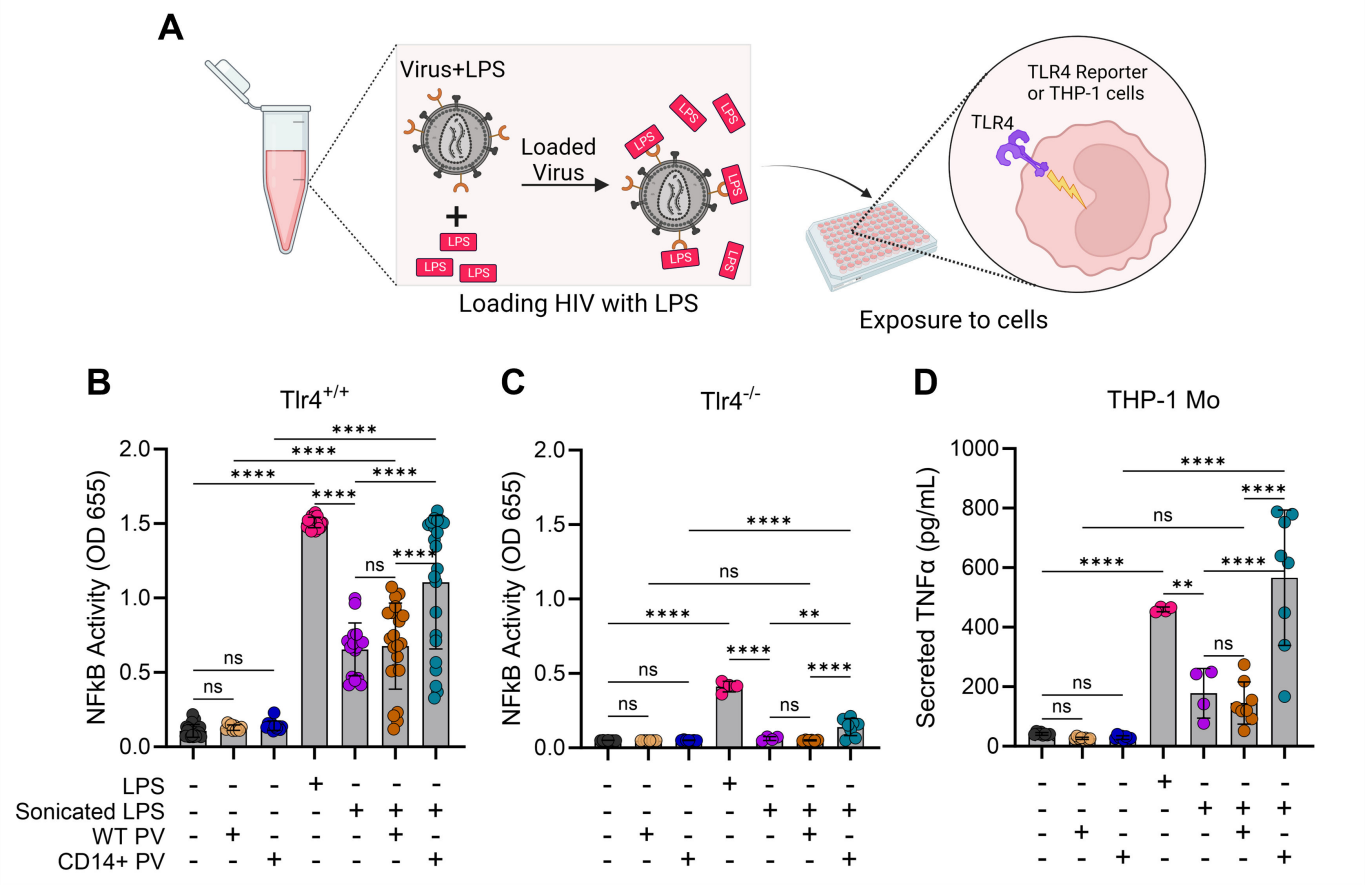
CD14^+^ HIV-1 can trigger TLR4 signaling and NF-κB activation by delivering LPS to human monocytes. (**A**) Schematic describing how virions were “loaded” with LPS before overlaying onto TLR4-expressing cells. (**B**) NF-κB was proxied by measuring SEAP activity from TLR4-expressing (Tlr4^+/+^) THP1-Dual cells induced after 24-h stimulation with samples. Bars are mean ± SD across four to five independent experiments, and where indicated, at least four different virus stocks were tested each time. Statistical significance was evaluated with one-way analysis of variance (ANOVA) (α = 0.05) and Tukey’s correction for multiple comparisons. From left to right: *n* = 21, *n* = 11, *n* = 12, *n* = 21, *n* = 16, *n* = 22, and *n* = 22. ns, non-significant; ****P < 0.0001. (**C**) Experiment as in (**B**) but done on TLR4-KO (Tlr4^-/-^) THP1-Dual cells. Data are mean ± SD of two independent experiments using four different virus stocks. Statistical significance was evaluated as in (**B**). From left to right: *n* = 7, *n* = 6, *n* = 7, *n* = 4, *n* = 4, *n* = 6, and *n* = 8. ns, non-significant; **P = 0.0014; ****P < 0.0001. (**D**) Secreted TNF-α was measured by ELISA from stimulated (24 h) THP-1 cells. Data are mean ± SD across two independent experiments with four different virus stocks. Statistical significance was evaluated with one-way ANOVA (α = 0.05) and Tukey’s correction for multiple comparisons. From left to right: *n* = 8, *n* = 8, *n* = 8, *n* = 4, *n* = 4, *n* = 8, and *n* = 8. ns, non-significant; **P = 0.0052; ****P < 0.0001.

On cells, LPS binding to CD14 is followed by LPS delivery to TLR4/MD-2, which then initiates two major intracellular signaling pathways, constituting the innate immune response to gram-negative bacteria (38, 51–53). As such, we wanted to determine if CD14^+^ HIV-1 can bind and deliver LPS to immune cells to initiate inflammatory signaling (Fig. 5A). To facilitate these studies, we turned to a commercially available TLR4 reporter cell line, THP1-Dual, derived from the THP-1 monocytic cell line. These cells express secreted alkaline phosphatase (SEAP) and secreted Lucia luciferase upon NF-κB and interferon (IFN) regulatory factor 3 (IRF3) activation, respectively. This model therefore allowed us to simultaneously assess both LPS-induced pathways after adding virions loaded with LPS.

Initially, we sought to mix purified LPS with our virions and centrifuge it through a size exclusion membrane to separate the unbound LPS from virion-bound LPS. During the optimization phase, we diluted LPS alone (Fig. S3A, Pre-filtration) and passed it through an Amicon column to obtain a post-filtration and filtrate fractions (Fig. S3A), which were then layered onto THP1-Dual cells to readout NF-κB activation (Fig. S3B). For this experimental design to work, we would expect most of the LPS to pass through the column and into the filtrate fraction. Unfortunately, this workflow proved unfeasible since a large fraction of the purified LPS remained either atop or bound to the membrane of the filter (Fig. S3B, Pre vs Post) and only a small fraction of LPS passed through the filter (Fig. S3B, Filtrate).

It became evident that the aggregation state of our LPS preparations would impede this experimental design, since aggregate size can vary greatly (54, 55). We then turned to sonication as it has been used to disaggregate LPS by others (56–58). The workflow (Fig. 5A) involved sonicating purified LPS and diluting it down to 50 ng/mL before adding it to WT or CD14^+^ HIV-1 virions. LPS-loaded virions were then overlayed onto TLR4 reporter cells and NF-κB activation was proxied by detecting SEAP enzymatic activity (Fig. 5B). Absorbance was negligible for cell controls (media only, black circles), WT PVs (tan circles) and CD14^+^ PVs (blue circles) added to cells in the absence of LPS, establishing a baseline NF-κB activity (Fig. 5B). As expected, purified LPS induced robust NF-κB activity (magenta circles) which was attenuated upon sonication (purple circles). The addition of WT virions to sonicated LPS did not affect NF-κB activity relative to sonicated LPS alone (orange vs purple). However, when CD14^+^ virions were incubated with sonicated LPS, there was a significant induction of NF-κB activity beyond sonicated LPS alone (teal vs purple), to levels similar to purified LPS (magenta). Matched TLR4 knockout (KO, Tlr4^-/-^) reporter cells were used to evaluate the specific involvement of TLR4 in these responses (Fig. 5C). Though NF-κB activity was observed to be largely TLR4-dependent (Fig. 5B vs C ), we detected a low level of TLR4-independent induction of NF-κB, particularly for purified LPS and LPS-loaded CD14^+^ HIV-1 (magenta and teal, respectively, Fig. 5C).

Having demonstrated that CD14^+^ HIV-1 virions can bind LPS and trigger an NF-κB response (Fig. 5B), we next determined if cytokines can be secreted downstream of the activated transcription factor. The prototypical inflammatory cytokine, TNF-α, is secreted by primary human monocytes and monocytic cell lines upon LPS-induced NF-κB activation (59–61). To that end, we conducted a similar experiment with LPS-loaded virions overlayed onto THP-1 monocytes and then assayed the culture supernatants for secreted TNF-α (Fig. 5D). Consistent with the trend for NF-κB activity (Fig. 5B), TNF-α secretion (Fig. 5D) was induced with purified LPS alone (magenta), attenuated with sonicated LPS (purple vs magenta), and restored when sonicated LPS was incubated with CD14^+^ (teal vs purple), but not WT (orange vs purple) HIV-1 virions. Thus, CD14^+^ virions can bind LPS and induce NF-κB activity, leading to inflammatory cytokine secretion.

### LPS-loaded virions can trigger TLR4 signaling through IRF3 activation

**Fig 6:**
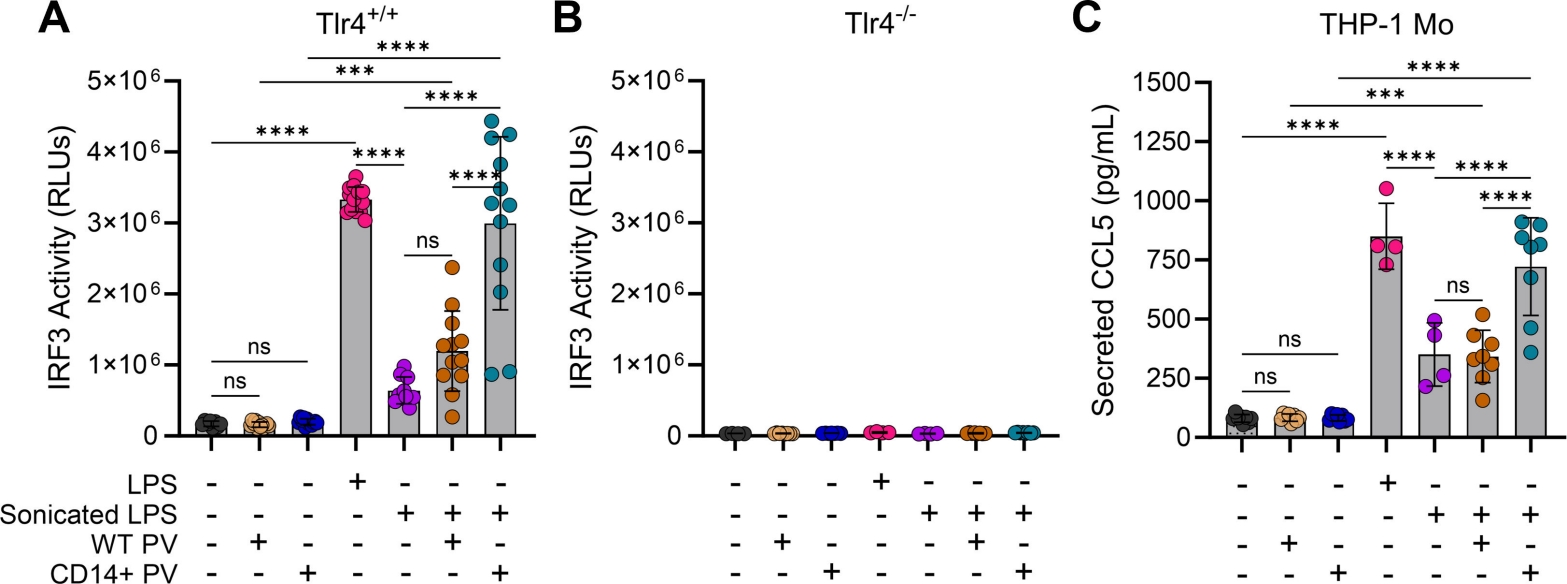
LPS-loaded virions can trigger TLR4 signaling through IRF3 activation. (**A**) IRF3 activation from TLR4-expressing (Tlr4^+/+^) THP1-Dual cells was measured by luciferase activity. Bars are mean ± SD of four different virus stocks across two to three independent experiments. Statistical significance was evaluated with one-way analysis of variance (α = 0.05) and Tukey’s correction for multiple comparisons. From left to right: *n* = 8, *n* = 12, *n* = 12, *n* = 14, *n* = 10, *n* = 12, and *n* = 12. ns, non-significant; ***P = 0.0003; ****P < 0.0001. (**B**) IRF3 activation from TLR4-KO (Tlr4^-/-^) THP1-Dual cells. Data are mean ± SD of two independent experiments using four different virus stocks. From left to right: *n* = 4, *n* = 8, *n* = 7, *n* = 4, *n* = 4, *n* = 8, and *n* = 8. (**C**) Secreted CCL5/RANTES was measured by ELISA from stimulated (24 h) THP-1 cells. Data are mean ± SD across two independent experiments with four different virus stocks. Statistical significance was evaluated as in (**A**). From left to right: *n* = 8, *n* = 8, *n* = 8, *n* = 4, *n* = 4, *n* = 8, and *n* = 8. ns, non-significant; ***P = 0.0005; ****P < 0.0001.

In addition to NF-κB, activation of TLR4 by LPS can also induce a second signaling pathway eliciting IRF3 activation (38, 52, 62). Using the THP1-Dual cells, we found that IRF3 activation followed a similar trend to NF-κB (Fig. 6A). Unlike for NF-κB, however, TLR4 was indispensable for IRF3 activation across all experimental conditions, as evidenced by the lack of luminescence observed with TLR4 KO cells (Fig. 6B).

We next assayed for IRF3-inducible cytokines to determine if IRF3 activation (Fig. 6A) can be substantiated with cytokine expression and secretion. The C-C chemokine CCL5 (or RANTES) is a gene target for IRF3 induced by LPS activation (38, 62, 63). CCL5 secretion was induced by LPS alone (Fig. 6C, magenta) and reduced by sonicated LPS (purple). Incubation of sonicated LPS with CD14^+^ virions (teal) but not WT virions (orange) increased the secretion of CCL5 relative to sonicated LPS alone (purple). Thus, CD14^+^ HIV-1 loaded with LPS can elicit both NF-κB and IRF3 activation, leading to cytokine secretion via both pathways.

Canonically, IRF3 activation is synonymous with type I interferon production (e.g., IFN-β), but this process is contingent on co-operative NF-κB activation as part of a larger enhanceosome complex that drives interferon-β gene induction (64–67). Therefore, having observed both NF-κB (Fig. 5A) and IRF3 (Fig. 6A) activation, we also assayed for IFN-β but were unable to detect any secreted IFN-β by THP-1 monocytes in response to LPS-loaded virions or even LPS alone (Fig. S4A). Given that THP-1 MDMs were previously shown to produce IFN-β more robustly than THP-1 monocytes in response to LPS (68), we tested THP-1 MDMs as well. As with THP-1 monocytes, THP-1 MDMs did not secrete IFN-β in response to LPS or loaded virions (Fig. S4B).

To confirm that both model systems had the capacity for IFN-β secretion, we transfected them with plasmid DNA (PolyJet + DNA) and observed the induction of IFN-β secretion (Fig S4E and F, purple circles), consistent with prior reports of double-stranded (ds) DNA triggering innate immune responses in these cells (69–71). Control conditions (transfection reagent alone, “PolyJet”) did not induce IFN-β secretion (Fig S4E and F, tan circles). Transfected DNA was also confirmed to induce NF-κB (Fig. S4C) and IRF3 (Fig. S4D) activation in THP1-Dual cells, suggesting that the observed IFN-β secretion by THP-1 cells (Fig. S4E and F) was IRF3-driven. Interestingly, transfection reagent alone was able to induce NF-κB but not IRF3 activation (Fig. S4 C vs D, “PolyJet”).

## DISCUSSION

As nascent virions bud from infected cells and acquire their viral envelope, they take with them a mosaic of human proteins decorating cell surfaces. This generates additional virus-associated proteins beyond what the limited viral genome encodes, and the functions of these added human proteins become imparted onto virions. For HIV-1, this phenomenon can significantly impact virus biology in a myriad of ways, including by more efficient attachment to target cells, enhancing infectivity, protection from complement-mediated viral lysis, and enhanced homing to gut mucosa where target cells are most abundant (11–22, 25). The present study introduces novel insight into the biological impact of virion-incorporated CD14. We first evaluated the presence of CD14 on lab-adapted strains of HIV-1, demonstrating widespread incorporation across common isolates grown in PBMCs and MDMs. These data support earlier observations detecting virion-incorporated CD14 in the plasma of HIV-infected patients (31–33).

Cellular CD14 is a GPI-anchored molecule with a well-characterized role in binding bacterial LPS. Therefore, incorporation of CD14 into the HIV-1 envelope is an observation with a range of biological consequences, considering that LPS binding is a first step in the innate immune recognition and response to gram-negative bacteria (38–40). By employing our optimized PV models, we aimed to understand the functional impact of CD14 incorporation by HIV-1. Leveraging our technical advances in using FVM to stain and phenotype virions (25, 36, 37), we tested if virions with incorporated CD14 could bind to a commercially available LPS-bio. We confirmed that CD14 incorporated into the HIV-1 envelope remained functional in its ability to bind LPS with FVM assays, which was corroborated by immunoprecipitation of virions with plate-bound, unlabeled LPS. While our earlier studies developed FVM protocols with direct labeling methods to phenotype proteins incorporated into viral envelopes, this work also offers several advancements in FVM methodology, as we now show that indirect fluorescence-labeling protocols can also permit efficient and reliable detection of antigens on the virion surface. In addition, we also demonstrated that antibody-mediated neutralization of biological activity can be measured with FVM, further enhancing the utility of FVM techniques through this study.

At the time of preparing this manuscript, exciting new work proposed that EVs bridge the intracellular sensing of extracellular microbial components to mediate innate immune responses (50). This revealed a potential limitation of our study since the authors highlighted the capacity of EVs to bind LPS through lipid-lipid interactions, prompting us to evaluate the specificity of the LPS binding we observed. Although the HIV-1 envelope is a phospholipid bilayer similar in composition to EV membranes, when we probed the specificity of LPS binding to virions, we observed no passive binding of LPS to WT virions that are devoid of incorporated CD14. Moreover, LPS binding to CD14^+^ virions was blocked by an antibody against CD14 in a dose-dependent manner. Collectively, these data refute the passive interaction between LPS and virions, indicating that LPS binding to virions depends on the presence of CD14 in the virion envelope.

LPS binding to cellular CD14 initiates a signaling cascade via TLR4 [reviewed elsewhere (38, 72)]. TLR4 signaling can follow either a MyD88- and TRIF-driven pathway, leading to the activation of transcription factors (e.g., NF-κB for MyD88; IRF3 for TRIF) for production of pro-inflammatory cytokines and type I interferons, respectively. In addition to demonstrating that CD14^+^ HIV-1 can bind LPS, we also provided novel evidence for the signaling capacity of these “LPS-loaded” virions. Viron-incorporated CD14 allowed HIV-1 virions to bind LPS and reverse the sonication-induced loss of LPS activity, which was not observed for WT virions. We showed that bioactive LPS loaded on CD14^+^ virions could activate NF-κB and IRF3 in a TLR4 reporter cell line, and in THP-1 monocytes, it triggered the secretion of TNF-α and CCL5. While others have described the impact of incorporated immunoregulatory proteins on cell signaling (26–28), our work highlights a pivotal role for CD14 in allowing virions to function as a shuttle for bioactive molecules like LPS and eliciting inflammatory responses.

When designing experiments to assess virus-mediated activation of signaling pathways, we wondered if there might be a MyD88-dependent signaling bias for NF-κB. We reasoned that the antiviral outcome of the TRIF-dependent (i.e., MyD88-independent) induction of IRF3 may be disadvantageous for the virus. However, results from the TLR4 reporter cell line indicated that both NF-κB and IRF3 get activated by CD14^+^ virions loaded with LPS. Activated NF-κB (59–61) and IRF3 (38, 62, 63), then, were presumably responsible for the secretion of TNF-α and CCL5, respectively, by THP-1 cells. This rationale was partially confounded by the absence of the canonical IRF3 gene target, IFN-β, being secreted by monocytes and macrophages tested herein. After confirming that both cell types retained the capacity to secrete IFN-β when challenged with transfected DNA, we posited that the amount of LPS used in this study (50 ng/mL) was likely insufficient to trigger IFN-β secretion by THP-1 monocytes, but paradoxically sufficient to elicit IRF3 activation and CCL5 secretion. Alternatively, MyD88-dependent IRF3 activation, though uniquely occurring (73–76), may explain the unexpected observation of IRF3 induction and the lack of IFN-β secretion. Additionally, such a phenomenon may also be explained by ineffective internalization of LPS bound to virions, since the TRIF/IRF3 pathway is initiated by endosomal TLR4 after internalization (38). While the presence of a signaling bias remains unclear, the molecular mechanisms underlying IRF3 activation by LPS-loaded virions will continue to be investigated.

Our current study reveals intriguing parallels with observations from mouse mammary tumor virus (MMTV), whereby virions from infected mice incorporated CD14 and TLR4 (43). The consequence of this incorporation was profound, as it highlighted a unique mechanism that allows MMTV to subvert immune responses. By binding to LPS from commensal gut microbiota, virions induced the immunosuppressive cytokine interleukin (IL)-10 which was important to tolerize infected mice (43, 77, 78). Wilks et al. have also noted that MMTV-incorporated antigens could potentiate TLR4 signaling by LPS from *Bacteroides thetaiotaomicron*. (43) Commensals from the *Bacteroides* genus are among the most abundant gram-negative species resident to the human gut (79), and possess LPS that are weak TLR4 agonists (80). Thus, we speculate that CD14 incorporation into HIV-1 may facilitate a similar interaction, highlighting the impact that incorporated host proteins may have on potentiating immune responses when challenged with otherwise agnostic stimuli. The significance of these findings stresses the need for more thorough investigations into the functional outcome of CD14 incorporation by HIV-1, especially given the intricate relationship between HIV-1, the human gut, and microbial translocation.

Microbial translocation is a process by which microbes/microbial products from the gut lumen cross the intestinal epithelial barrier and enter the systemic circulation (81). HIV-1 infection is associated with changes in the gut microbiome and epithelia integrity, which may contribute to disease progression and immune dysfunction. Specifically, the loss of tight junction proteins in the intestinal epithelial barrier have been linked to microbial translocation, increased immune activation, and increased systemic inflammation (82–84). Several mechanisms have been proposed for microbial translocation, including dysbiosis, depletion of mucosal CD4^+^ T cells and pro-inflammatory cytokine production by innate immune cells (81, 82). This broader contextualization emphasizes the need for understanding CD14 incorporation by HIV-1 within the intricate landscape of HIV-associated gut pathology and microbial translocation, insofar as HIV may bind LPS from translocated microbes and perpetuate gut inflammation. Notably, however, chronic inflammation is experienced even when HIV-1 viral load is suppressed with antiretroviral therapy (29). This suggests that any impacts of virion-incorporated CD14 on inflammation may be limited in this context or, alternatively, restricted to instances where viral load is detectable.

CD14 has long been implicated in the pathogenesis of HIV infection. Numerous studies have identified plasma levels of soluble CD14 (sCD14) as a prognostic indicator for rapid disease progression (85), increased mortality (86), and other adverse clinical outcomes (87–93) in HIV-infected cohorts. However, a clear role or direct cause of elevated sCD14 in HIV infection remains to be defined. It is plausible that plasma CD14 is merely indicative of immune activation and systemic inflammation, which would be consistent with similar observations of elevated sCD14 in diseases unrelated to HIV infection (94–97). However, the impact of virion-incorporated CD14, described in the present study along with previous work (31–33), warrants consideration that CD14 detected in the plasma of HIV-infected patients may be a combination of inflammation-related sCD14 and also virion-bound CD14.

In conclusion, our study sheds light on new potential roles for CD14 incorporation by HIV-1, particularly in the context of immune modulation. The incorporation of CD14 into the HIV-1 envelope not only allows for efficient viral binding of LPS, but can also enable viruses to trigger LPS-mediated signaling pathways, leading to the secretion of pro-inflammatory cytokines by immune cells. Coupled with the chronic gut inflammation experienced by HIV-infected individuals, this study provides a credible rationale for future studies to explore the contributions of virion-incorporated CD14 to HIV-1 disease pathogenesis.

## MATERIALS AND METHODS

### Cell lines and culture reagents

Prior to experiments, all cells (primary and cell lines) were enumerated for counts and viability on the Muse Cell Analyzer using the Muse Count and Viability Kit (Luminex). All cells were incubated in a humidified 5% CO_2_ incubator at 37°C, unless otherwise specified. RPMI-1640 (#350-000CL), Dulbecco’s modified Eagle medium (DMEM; #319-005 CL), fetal bovine serum (FBS, heat inactivated at 56°C for 30 min; #098150), and trypsin/EDTA (#325-043-CL) were all obtained from Wisent. Purified unconjugated LPS (*Escherichia coli* serotype O55:B5) was obtained from Sigma-Aldrich (#L6529). Normocin (#ant-nr-1), blasticidin (#ant-bl-05), Zeocin (#ant-zn-05), and LPS-bio (*E. coli* O111:B4, #tlrl-lpsbiot) were obtained from Invivogen. PE-SA (#12-4317-87), LPS-AF488 (*E. coli* O55:B5, #L23351), heparinized vacutainers (#02-685-3B), Opti-MEM (#31985070), and penicillin/streptomycin (Thermo Fisher #15140122) were obtained from Thermo Fisher Scientific. rLBP (#870-LP-025) and phorbol 12-myristate 13-acetate (PMA, #1201) were obtained from R&D Systems. Lymphoprep was obtained from Stem Cell Technologies (#07861).

293T cells used in transfections were obtained from the American Type Culture Collection (ATCC) and maintained in complete media comprising DMEM, 10% FBS, 100 U/mL penicillin, and 100 µg/mL streptomycin. 293T cells were trypsinized and passaged every 2–3 days in T75 flasks and maintained to a maximal 80%–85% confluency.

The THP-1 cell line was obtained from ATCC and maintained in complete media comprising RPMI-1640, 10% FBS, 100 U/mL penicillin, and 100 µg/mL streptomycin. Cultures were passaged every 2–3 days in T75 flasks and maintained to a maximal density of 1 × 10^6^ cells/mL. To differentiate THP-1 into MDMs, THP-1 cells were plated in 96-well plates (1 × 10^5^ cells/well) in 200 µL complete RPMI and treated with 10 ng/mL PMA. After 48 h in PMA, adherent cells were washed with RPMI ×3 and allowed to incubate for an additional 48 h before experimentation.

The THP1-Dual reporter (Tlr4^+/+^, #thpd-nfis) and the matched TLR4 knockout (Tlr4^-/-^, #thpd-kotlr4) cell lines were obtained from Invivogen. Cells were maintained as described by the manufacturer in complete RPMI supplemented with 100 µg/mL Normocin. Both Dual cell lines were passaged every 3 days with antibiotic selection every other passage (10 µg/mL blasticidin and 100 µg/mL Zeocin). For all experiments involving THP-1, THP-1 MDM, and THP1-Dual, cells were plated in Opti-MEM supplemented with 2% FBS, 100 U/mL penicillin, 100 µg/mL streptomycin. For THP-1 MDMs, conditioned media post-differentiation was removed and replaced with Opti-MEM.

### Primary cell culture

PBMCs were isolated by density centrifugation by first diluting blood twofold with 2% FBS + PBS and layering over Lymphoprep in a 3:1 ratio of diluted blood to Lymphoprep. Overlays were centrifuged (800 × *g* for 25–35 min) at room temperature with the brake off, and the PBMC fraction was removed. PBMCs were washed twice with 2% FBS + PBS and centrifugation at 200 × *g* to reduce platelet contamination and washed again at a centrifugation speed of 300 × *g*. After this step, PBMCs were counted and infected for primary virus production, or used to isolate myeloid cells for differentiation into MDMs.

To obtain MDMs, PBMCs were resuspended in complete media comprising RPMI-1640, 10% FBS, 100 U/mL penicillin, and 100 µg/mL streptomycin. Cells were added to Nunc T75 flasks (Thermo Fisher #156499) at 5–7 × 10^6^ cells per flask in 10 mL and incubated for 1–2 h to allow myeloid cells to adhere. Non-adhered cells (mostly lymphocytes) were removed by extensive washing (5× with RPMI), and adherent cells (mostly monocytes) were allowed to incubate for 6 days in complete RPMI supplemented with donor-matched plasma (1:10). After day 6, MDMs were used for infection to propagate macrophage-tropic primary HIV-1 isolates.

### Virus culture in primary cells

PBMC and MDMs were obtained as described above. For PBMC infection, cells were centrifuged and resuspended in 1 mL undiluted HIV-1_IIIB_, HIV-1_NL4-3_, HIV-1_BaL_, and HIV-1_SF162_ (BEI Resources, formerly the NIH HIV Reagent Program) for 4 h at 37°C. Afterward, infected cells were transferred into T75 flasks with fresh complete RPMI (described above) and incubated until the time of harvest (7–12 days post infection, dpi). Fresh media were occasionally added throughout the infection to ensure optimal cell confluency. On the day of harvest, cell culture supernatants containing virus were centrifuged to remove the cells, after which it was aliquoted and stored at −80°C until use.

For MDM infections, conditioned media after 6 days of differentiation (above) were removed and cells were washed twice with PBS (with Ca^2+^/Mg^2+^) and then once with complete RPMI. Then, 2 mL of a 1:1 mixture comprising RPMI and either HIV-1_BaL_ or HIV-1_SF162_ (National Institutes of Health AIDS Reagent Program) was added to monolayers for 2 h at 37°C, rotating the flasks twice during that time to ensure inoculum remained evenly distributed. After inoculation, fresh RPMI (10–12 mL) was added to each flask and cells were incubated until 18 dpi, after which virus was harvested and frozen, as described above.

### Pseudovirus production

PV was generated by co-transfecting plasmids encoding an Env-deficient HIV-1 backbone (pSG3ΔEnv, NIH ARP #11051), CD14 (pCD14, AddGene #13645), and/or the HIV-1 envelope glycoprotein (pBaL.01, NIH ARP #11445) into 293T cells. In brief, cells were plated at 3 × 10^5^ cells/well in 12-well plates (1 mL complete DMEM) and allowed to adhere overnight. The next morning, plasmid DNA was diluted and mixed with Poly Jet transfection reagent following the manufacturer’s instructions (Frogga Bio #SL100688). For WT PVs, 0.5 µg SG3ΔEnv plasmid was transfected alone, and for CD14^+^ PVs, 0.5 µg SG3ΔEnv + 0.25 µg CD14 plasmids were co-transfected. Cells were cultured for 48 h after transfection, after which cell culture supernatants containing virus particles were collected, centrifuged to remove cellular debris, and frozen at −30°C until use.

### Flow virometry assays

For staining, cell-free supernatants containing virus particles were combined 1:1 with PE-conjugated anti-CD14 (mAb M5E2, BD Biosciences #561707), isotype-matched control (mAb MOPC-173, BD Biosciences #565363), LPS-AF488, or LPS-bio diluted in PBS at the concentrations specified in text. For the LPS-bio samples, a secondary staining step was performed with PE-SA, incubated 1:1 at the specified concentrations for 4 h at 4°C. Here, the “specified concentrations” refer to the final staining concentrations stated in the figure legends, acknowledging that mAbs, LPS-bio,and PE-SA were diluted to 2× for the 1:1 incubation with virus samples. See Results for caveats of using LPS-AF488 in flow virometry assays.

Where specified, mAbs were serially diluted twofold from 1.6 to 0.1 µg/mL for determination of antibody titration curves; LPS-bio and PE-SA were diluted from 600 to 5 ng/mL and 1.6 µg/mL–0.05 µg/mL, respectively, for checkerboard titration; and PE-SA incubation was assessed over a 16-h time course. Where indicated, LPS-bio was pre-incubated with increasing concentrations of rLBP (1 µL; R&D Systems #870-LP-025) or purified anti-CD14 (1 µL; mAb M5E2, BD Biosciences #557152) before adding to samples overnight and subsequently developing with PE-SA the next day.

Before acquisition, all stained samples were further diluted in PBS (500- to 1,000-fold) to reduce coincidence and in lieu of washing. Samples were acquired on a CytoFLEX S equipped with CytExpert v.2.4 (Beckman Coulter) with standard optical configuration at 10 µL/min for 30–60 seconds (25, 36, 37). Data were acquired and analyzed in accordance with MIFlowCyt-EV framework for standardized reporting and analysis of small particles by cytometry (34, 35). Gain and threshold optimization for the detection of virions were done as previously described (35). BD Quantibrite PE beads (BD Biosciences #340495, lot 91367) and NIST-traceable 3000 Series Nanosphere size standards (Thermo Fisher #3020A) were used for fluorescence and light scatter calibrations, respectively. Data calibration was done using the FCM_PASS_ software (https://www.fcmpass.com) (45, 98). Detailed calibration parameters are noted in the FCM_PASS_ report (Table S1) and the MIFlowCyt-EV checklist (34) appended (Table S2).

Data analysis after calibration was done on Flow Jo v.10.10.0. Median PE MESF values and particle counts were extracted from stained samples in Flow Jo using the gating strategy specified in text. Gates demarcating virus populations and PE^+^ events on pseudocolor dot plots were defined using antibody-alone controls (35). For titration data, a stain index calculation was performed using the following formula to determine optimal concentrations:


$$Stain\;index=\;\frac{FL{\;}_{POS}\;-\;FL{\;}_{NEG}}{2\;\times \;SD_{\;NEG}},$$

where *FL_POS_* and *FL_NEG_* are the quantitative fluorescence units (MESF) of the positive and negative populations, respectively, and *SD_NEG_* is the robust standard deviation of MESF value of the negative population. For antibody titration, the negative population was defined as PE^+^ events from the isotype control stain, whereas the positive population was defined as the PE^+^ events stained by anti-CD14-PE. For LPS-bio and PE-SA titrations, the negative population was set to the instrument’s optical noise and the positive population was set to the PE^+^ events for each PE-SA stain.

### Virion capture assays

Virion capture (plate-bound immunoprecipitation) assays (Fig. 1A) were done using either mAbs or bacterial LPS. LPS from *E. coli* O55:B5, anti-CD14 (mAb M5E2, BD Biosciences # 561707), anti-gp120 (mAb PG9, NIH ARP #12149), or isotype-matched control monoclonal antibodies (all at 5 µg/mL diluted in PBS) were adsorbed onto 96-well plates (Nunc MaxiSorp, Thermo Fisher #12-565-136) overnight at room temperature. Following three consecutive PBS washes, wells were blocked for 1 h at room temperature (5% bovine serum albumin/PBS). Virus samples were diluted as necessary to equalize the input for samples with differing amounts of Gag_p24_, added to wells, and allowed to interact with bound antibodies overnight at 4°C. To detect captured virus, wells were washed three times with PBS and treated with lysis buffer (0.5% Triton in PBS) to quantify the HIV-1 capsid protein, Gag_p24_, with a high-sensitivity AlphaLISA (Perkin Elmer).

### Western blotting

HIV-1_BaL_ and HIV-1_SF162_ from infected MDMs (two donors each) and HIV-1_IIIB_ and HIV-1_NL4-3_ from infected PBMCs (two and four donors, respectively) were concentrated using PEG (PEG-It, System Biosciences), and resuspended in radioimmunoprecipitation assay (RIPA) buffer (BioBasic #RB4478) to achieve viral lysates that are ~50×–60× more concentrated than the starting stocks comprised of virus culture supernatants. Donors for each isolate were pooled together before PEG concentration. After quantitation of the HIV-1 capsid protein (Gag_p24_) in RIPA lysates for normalization of virus input, samples were denatured in Laemmli buffer and boiled for 10 min at 95°C. For SDS-PAGE, 30 ng of p24 was separated on an 8% resolving gel (Tris-Glycine-SDS buffer, BioRad #1610772) and transferred to polyvinylidene fluoride (PVDF) membranes (Pierce methanol-free transfer buffer, Thermo Fisher #35045). Membranes were blocked with 5% skim milk/Tris-buffered saline-Tween (TBST; 0.1% Tween-20) for 1 h, with shaking at room temperature. Primary antibodies were diluted in TBST and added overnight at 4°C: human anti-HIV gp120 (mAb 39F, 0.15 µg/mL, NIH ARP #11437), mouse anti-HIV gp120 (mAb B23, 1:5, gifted from G. Lewis), rabbit anti-human CD14 (mAb ARC54507, 0.2 µg/mL, ABclonal #A21256), and mouse anti-HIV p24 (mAb AG3.0, 1:1,000, NIH ARP #4121). The following day, membranes were washed three times with TBST and incubated with appropriate horseradish peroxidase (HRP)-conjugated secondary antibodies diluted in TBST for 1 h at room temperature (RT): goat anti-human IgG (1:10,000, Sigma Aldrich #AP112P), goat anti-mouse IgG (1:10,000, Frogga Bio #BML-SA204-0100), and goat anti-rabbit IgG (1:10,000, Frogga Bio #ADI-SAB-300-J). After washing, membranes were developed using enhanced chemiluminescence and imaged on the ChemiDoc XRS+ (BioRad).

### Flow cytometry

Flow cytometry was performed where indicated on phenotype primary cells or transfected cell lines to confirm transfection efficiency or LPS-conjugate binding. For each stain, 0.5–1 × 10^6^ cells were centrifuged and washed with FACS buffer (2% FBS in PBS) before resuspending in 2 µg/mL PE-conjugated mouse anti-human CD14 (mAb M5E2, BD Biosciences #561707) or 100 ng/mL of LPS conjugates (Alex Fluor 488 or biotin). Where indicated, rLBP was pre-incubated with LPS for 10 min at 37°C prior to incubation with cells for binding. Cells were then washed in FACS buffer, and LPS-bio was detected using PE-SA (0.6 µg/mL, Thermo Fisher #12-4317-87). After washing, data were acquired on the CytoFLEX S using CytExpert v.2.4 (Beckman Coulter) and analyzed using Flow Jo v.10.10.0.

### Reporter cell assays, NF-κB and IRF3 activation, and cytokine ELISA

THP1-Dual reporter cells (Tlr4^+/+^ and Tlr4^-/-^) and THP-1 monocytes were plated in 96-well plates at 1 × 10^5^ cells/well in 150 µL Opti-MEM supplemented with 2% FBS, 100 U/mL penicillin, and 100 µg/mL streptomycin. THP-1 MDMs were generated as described above; on the day of stimulation, conditioned medium was removed and replaced with 150 µL Opti-MEM.

All samples used to stimulate THP-1 Dual cells were diluted in serum-free DMEM, unless specified otherwise. Purified LPS (*E. coli*, O55:B5) was diluted to 500 ng/mL, sonicated for 30 min (35 kHz/48W), and then diluted to the stimulating concentration of 50 ng/mL before sonication again. At this point, WT or CD14^+^ virions were added to sonicated LPS at 15 ng Gag_p24_ (measured by AlphaLISA) per reaction and incubated for 30 min at 37°C followed by 1–2h at 4°C. To induce IRF3 by DNA transfection, 100 ng DNA/well (CMV3 Negative Control Vector, Sino Biological #CV011) was mixed with Poly Jet and incubated for 15 min at room temperature. A Poly Jet-only control was also prepared without any DNA.

LPS-loaded virions were added to cells at 50 µL/well alongside positive controls (LPS alone and Poly Jet + DNA) and negative controls (serum-free DMEM, virus alone, and Poly Jet alone). After a 24-h incubation, cell-free supernatants from THP1-Dual cells were collected and assayed for SEAP (NF-κB reporter) and luciferase (IRF3 reporter) activity by QUANTI-Blue (Invivogen, #rep-qbs) and QUANTI-Luc (Invivogen, #rep-qlc4lg1), respectively. Absorbance (OD 655 nm) and luminescence were measured on the Synergy Neo 2 multi-mode plate reader equipped with Gen 5 v.5.01 (BioTek). For THP-1 monocytes and MDMs, cell-free supernatants were collected and assayed by DuoSet sandwich enzyme-linked immunosorbent assay (ELISA; R&D Systems) for secreted TNF-α (#DY210), CCL5 (#DY278), and IFN-β (#DY285B).

For size exclusion filtration, LPS alone (*E. coli*, O55:B5) was diluted to 50 ng/mL or 300 ng/mL in PBS or serum-free DMEM before passing through an Amicon column (100 kDa molecular weight cutoff [MWCO]; Sigma Aldrich #UFC510024) according to the manufacturer’s instructions. Fractions were collected and added to THP1-Dual cells and assayed for SEAP activity as a proxy for NF-κB induction as described above.

### Statistical analyses

All graphs and statistical analyses were done on GraphPad Prism v.10.1.2. For all data acquired with THP1-Dual cells (Tlr4^+/+^, optical density [OD] 655 nm, and luminescence), data were generated using at least four independently produced virus stocks across three to five independent experiments. To control for the inter-assay variability contributed by performing the assays on different plates on different days, data were normalized to positive controls after first confirming that the trends of each experimental replicate were similar. For normalization, the following formula was used:


$$Y_{Norm}=Y_{a}\;/\;\left(\frac{POS_{a}}{POS_{Ref}}\right),$$

where $Y_{Norm}$ is the transformed data, $Y_{a}$ is the raw data from a given plate per experiment, ${POS}_{a}$ is the positive control from the same plate per experiment, and $POS_{Ref}$ is the positive control from the reference plate which was arbitrarily assigned. All data sets from THP1-Dual (Tlr4^+/+^ and Tlr4^-/-^) were then tested for outliers using the robust regression and outlier removal (99) function in Prism. Where indicated, one-way and two-way analyses of variance (α = 0.05) were done with Tukey’s correction for multiple comparisons to infer statistical significance.

## Data Availability

FCS files for flow cytometry and flow virometry data sets are publicly available on FlowRepository.org under the identifier FR-FCM-Z76D.

## SUPPLEMENTAL MATERIAL

Figures S1 to S4. jvi.00363-24-s0001.pdf Optimizations of experimental methods.

Table S1 jvi.00363-24-s0002.pdf FCMpass output report.

Table S2 jvi.00363-24-s0003.pdf MIFlowCyt-EV checklist.

ASM does not own the copyrights to Supplemental Material that may be linked to, or accessed through, an article. The authors have granted ASM a non-exclusive, world-wide license to publish the Supplemental Material files. Please contact the corresponding author directly for reuse.
