# Supplementary material for: Virion-incorporated CD14 enables HIV-1 to bind LPS and initiate TLR4 signaling in immune cells
Source: J Virol. 2024 Apr 25;98(5):e00363-24. doi: 10.1128/jvi.00363-24 (PMC11092368; doi:10.1128/jvi.00363-24)
Supplement: Figures S1 to S4. — Optimizations of experimental methods. [file jvi.00363-24-s0001.pdf]

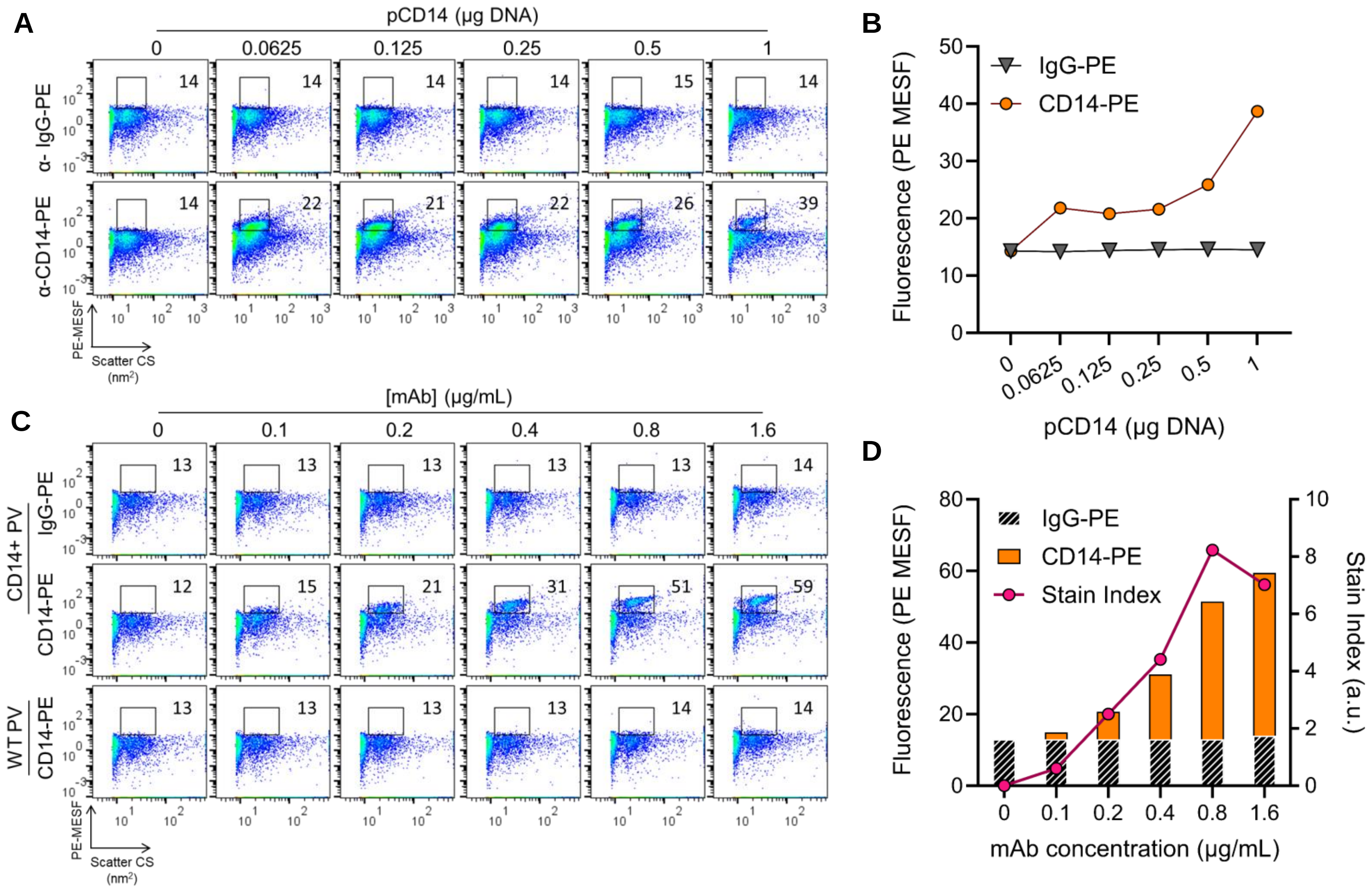

Fig S1. Optimizing the production and staining of HIV-1 pseudovirus models for CD14 incorporation. **(A)** Varying amounts of CD14 plasmid DNA (pCD14) were co-transfected with the HIV-1 backbone (pSG3ΔEnv) to produce virions with differing amounts of CD14 on the surface. Virions were then stained with PE-conjugated isotype control (top row) or anti-CD14 mAb M5E2 (bottom row) before FVM analysis. Gates demarcate PE^{+} events set relative to the isotype control. PE MESF values are enumerated for PE^{+} events. Data is representative of two independent transfections. **(B)** PE MESF values from (A) were plotted over the range of pCD14 tested for isotype control (IgG-PE, grey inverted triangle) and anti-CD14 (CD14-PE, orange circles) stains. **(C)** Titrating mAbs by FVM. PE-conjugated anti-CD14 mAb M5E2 (bottom two rows) or isotype control (top row) were titrated on CD14^{+} (top two rows) and WT virions (bottom row). Gates are the PE+ events and the associated PE MESF values are enumerated on each plot. Data are representative of two different titrations done using two different virus stocks. WT and CD14^{+} virus stocks shown in FVM plots had a respective titer of 224 and 132 ng/mL p24 as measured by ELISA. **(D)** PE MESF (left Y-axis) values for isotype control (IgG-PE, hatched bars) and anti-CD14 (CD14-PE, orange bars) stains from CD14^{+} PVs in (C) were plotted over the range of antibody concentrations tested. Stain index (magenta, right Y-axis) was calculated using IgG-PE and CD14-PE MESF values to identify optimal mAb concentration.

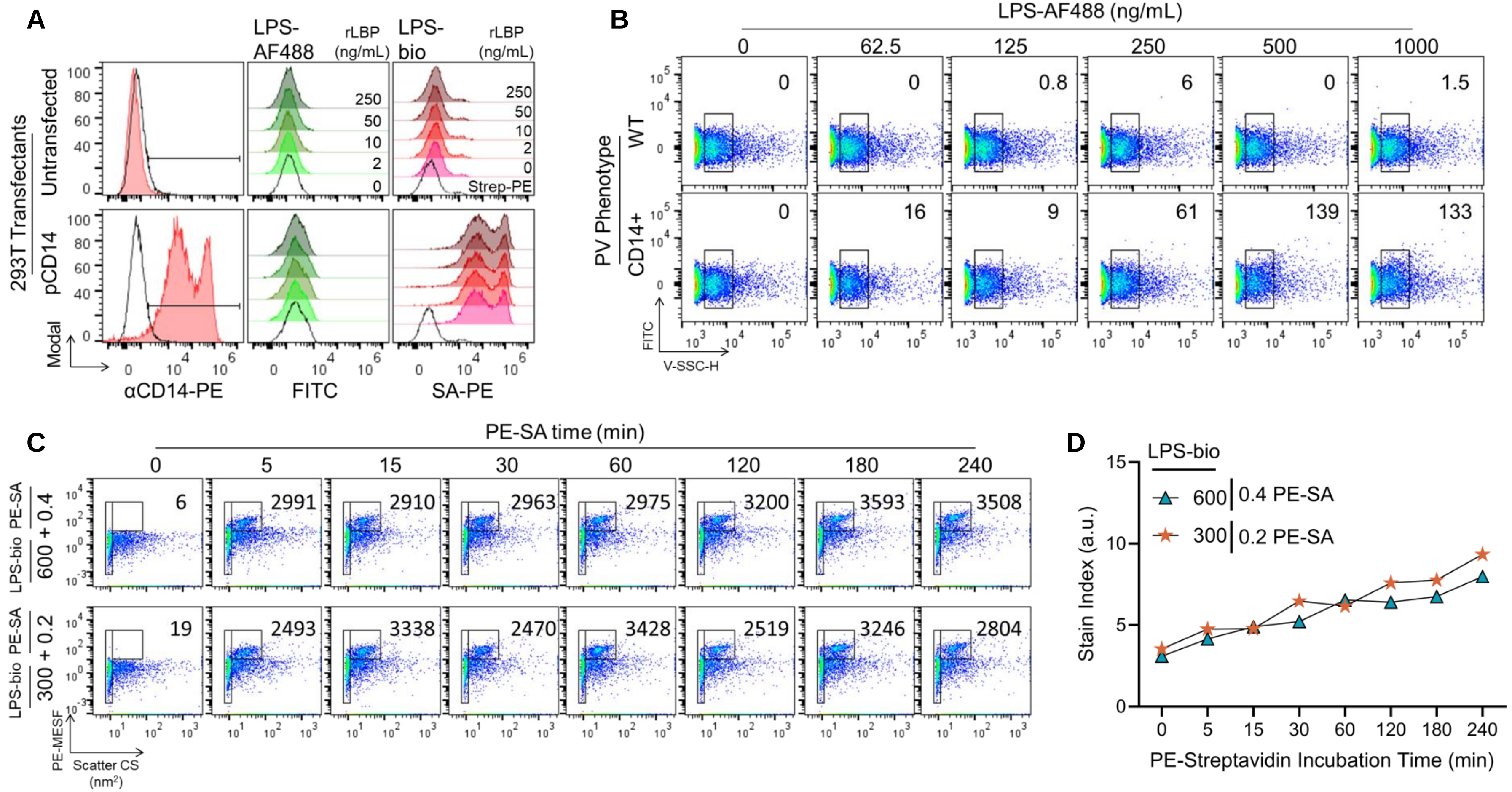

Fig S2. LPS-bio and PE-SA offers a highly sensitive approach for detecting LPS binding to virions. **(A)** Validating the binding of LPS-AF488 (middle column) and LPS-bio (right column) to CD14-transfected (bottom row) and untransfected (top row) 293T cells by flow cytometry, under different recombinant LBP concentrations (0-250 ng/mL). Cells were stained with PE-conjugated anti-CD14 (mAb M5E2, pink histograms) or isotype control (black histograms) to confirm CD14 expression (left column). LPS-bio was detected with PE-conjugated streptavidin (SA-PE). **(B)** FVM was used to evaluate the binding of LPS-AF488 (0-1000 ng/mL) to WT (top row) and $CD14^+$ virions (bottom row). Gates were drawn to include the entire virion population to better average the fluorescence intensity, since staining was less profound than seen for mAbs and LPS-bio (C). Mean fluorescence intensities (FITC) of the gated populations are enumerated. Data is representative of two independent virus stocks. **(C)** FVM was used to evaluate the binding of LPS-bio to $CD14^+$ virions over a range of incubation times with PE-SA (0-240 min). Two pairs of LPS-bio (ng/mL) and PE-SA (μg/mL) concentrations were tested (top vs bottom rows). Left gates indicate optical noise used in (D) and right gates define PE^+ events with enumerated particle counts values. WT and $CD14^+$ virus stocks shown in FVM plots of B-C had a respective titer of 45 and 73 ng/mL p24 as measured by ELISA. **(D)** Stain index calculations using the PE MESF of PE^+ particles in (C) were plotted over the PE-SA incubation times for each pair of concentration (blue triangles vs orange stars). LPS-bio and PE-SA are in units of ng/mL and μg/mL, respectively.

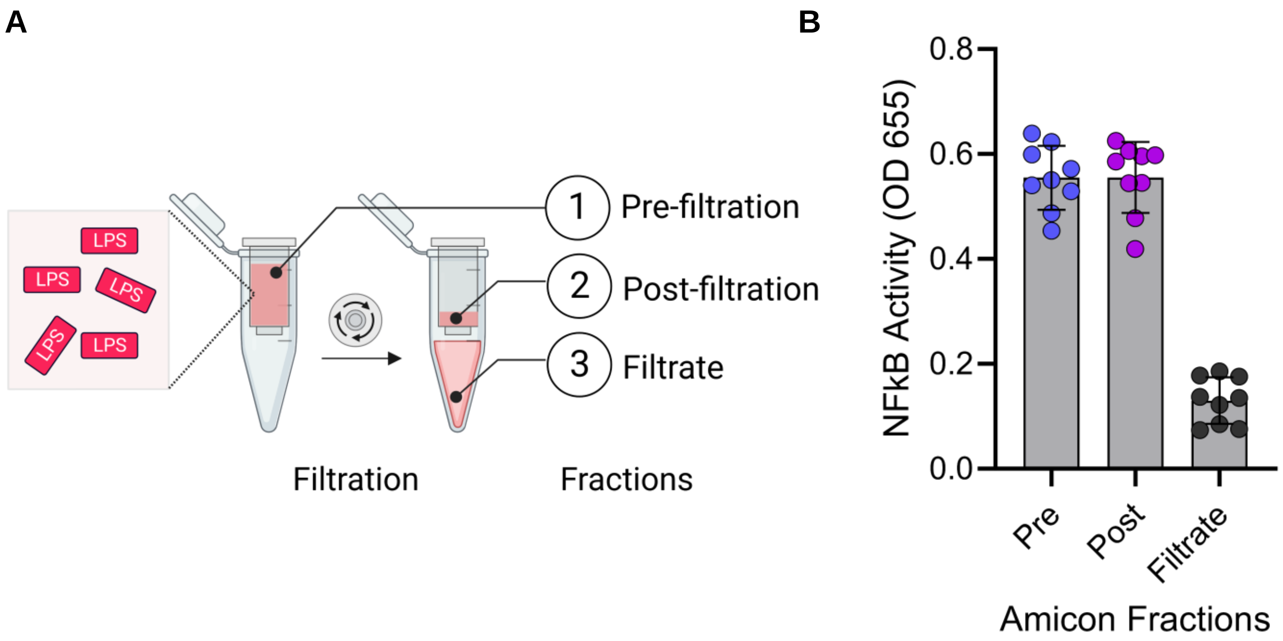

Fig S3. Determining the feasibility of size exclusion filtration on LPS. **(A)** Schematic depicting the sample preparation. LPS was diluted and added to an Amicon column (100 kDa MWCO) for size exclusion filtration. Fractions were collected before filtration ('Pre-filtration') and after filtration ('Post-filtration'), in addition to the flow-through ('Filtrate'). **(B)** Fractions collected in (A) were added to THP1-Dual cells and NF-κB activation was measured by SEAP enzymatic activity in supernatants. Data are mean ± SD of n=9 column filtrations.

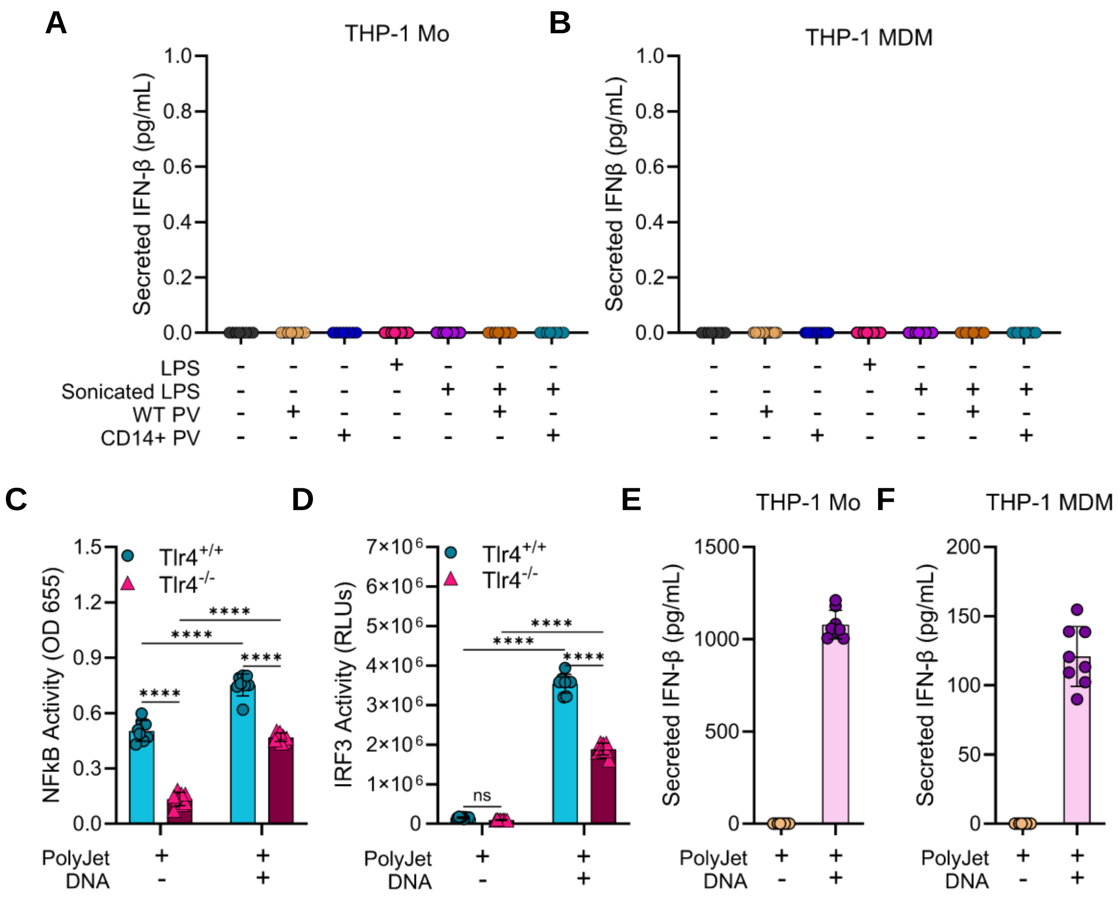

Fig S4. Determination of IFN-β response in THP-1 and PMA-differentiated THP-1. **(A)** Experiments were designed as in Figures 5-6. Secreted interferon-beta (IFN-β) was measured by ELISA from stimulated (24h) THP-1 monocyte (THP-1 Mo) supernatants. Data are mean ± SD of 2-3 independent experiments done using four different virus stocks each time. **(B)** As in (A) using monocyte-derived macrophages generated by PMA differentiation of THP-1 (THP-1 MDM). **(C)** NF-κB was proxied by measuring SEAP activity from TLR4-expressing (teal circles, $Tlr4^{+/+}$) or TLR4-KO (magenta triangles, $Tlr4^{-/-}$) THP1-Dual cells induced after 24h stimulation with transfected DNA or transfection reagent alone (Poly Jet). **(D)** IRF3 activation was measured by luciferase activity under the same conditions as in (C). For C-D, data are mean ± SD of two independent experiments done using four different virus stocks each time. Statistical significance was evaluated with two-way ANOVA ($\alpha = 0.05$) and Tukey's correction for multiple comparisons. n=8; ns, non-significant; **** $p<0.0001$. Secretion of IFN-β by THP-1 Mo **(E)** and THP-1 MDM **(F)** after 24h stimulation with transfected DNA or transfection reagent alone (Poly Jet). Data are mean ± SD of four different virus stocks across two independent experiments.

SUPPORTING INFORMATION

Fig. S1. Optimizing the production and staining of HIV-1 pseudovirus models for CD14 incorporation.

Fig. S2. LPS-bio and PE-SA offers a highly sensitive approach for detecting LPS binding to virions.

Fig. S3. Determining the feasibility of size exclusion filtration on LPS.

Fig. S4. Determination of IFN-β response in THP-1 and PMA-differentiated THP-1.

Table S1. (A) FCM_{PASS} output report for the acquisition parameters of samples, and fluorescence and light scatter calibration. (B) Exported FCM_{PASS} plots for light scatter and fluorescence calibration.

Table S2. MIFlowCyt-EV checklist in compliance with the MIFlowCyt-EV framework for standardized reporting of nanoscale cytometry.
