# Supplementary material for: Virion-incorporated CD14 enables HIV-1 to bind LPS and initiate TLR4 signaling in immune cells
Source: J Virol. 2024 Apr 25;98(5):e00363-24. doi: 10.1128/jvi.00363-24 (PMC11092368; doi:10.1128/jvi.00363-24)

A: FCMPASS output report for the acquisition parameters of samples, and fluorescence and light scatter calibration.

Flow Cytometer Modeling Settings

FCMPASS Version 4.2

SSC Parameter	Wavelength	Sheath RI	Determine HA	Aperture Geom	Theta	Phi	Eps	Cali. Factor (nm^2)
SSC_1-H | Violet SSC-H	405	1.3430917	on	circle	90	90	54.7	0.004105378

Light Scatter Calibration Reference Beads

Diameter (nm)	Diameter CV (%)	Measured RI	Measurement Wavelength	Composition	Acquired Stat	Acquired CV	Input	Acquisition Wavelength	Modelled RI	Manufacturer	Catalogue No.	Lot No.
81	11.7	1.59	589	Polystyrene	6435	11.7	Manual	405	1.625265271	ThermoFisher Scientific	3080A	228748
100	7.8	1.59	589	Polystyrene	20889	7.8	Manual	405	1.625265271		3100A	204935
152	3.3	1.59	589	Polystyrene	138043	3.3	Manual	405	1.625265271		3150A	202026
203	2.6	1.59	589	Polystyrene	394220	2.6	Manual	405	1.625265271		3200A	205131
269	1.6	1.59	589	Polystyrene	912000	1.6	Manual	405	1.625265271		3269	202729
303	1.6	1.59	589	Polystyrene	1160000	1.6	Manual	405	1.625265271		3300A	204665
345	1.9	1.59	589	Polystyrene	1380000	1.9	Manual	405	1.625265271		3350A	199283
401	1.3	1.59	589	Polystyrene	1520000	1.3	Manual	405	1.625265271		3400A	203859
453	1.7	1.59	589	Polystyrene	1800000	1.7	Manual	405	1.625265271		3450A	204047
480	4.2	1.45	589	Silica	258839	4.2	Manual	405	1.46110339		8050	203277
730	4.1	1.45	589	Silica	1040000	4.1	Manual	405	1.46110339		8070	207434

Fluorescence Calibration

Parameter	New Parameter Name	Reference Fluor	Reference Value 1	Reference Value 2	Reference Value 3	Acquired Value 1	Acquired Value 2	Acquired Value 3	F/P Ratio	Slope	Intercept	R-Square	Regression Type	Manufacturer	Cat. No.	Lot No.
FL9-A | PE-A	PE MESF	PE	474	5359	23843	73309	896000	3870000	1	1.01423414	2.156492534	0.999812322	weighted log	Beckton Dickinson	340495	47973

Sample Acquisition Information

The following extrapolations of thresholds to standard units assume that samples were acquired at the same detector settings as their calibration controls.

Filename	Sample Type	Trigger Parameter	Trigger ID	Trigger Threshold (au)	Detector Setting (au)	Trigger Threshold | Polystyrene (nm)	Trigger Threshold | Silica (nm)	Trigger Threshold | EV Diameter (nm) [High RI]	Trigger Threshold | EV Diameter (nm) [Average RI]	Trigger Threshold | EV Diameter (nm) [Low RI]	Trigger Threshold | rEV CS Diameter (nm)	Trigger Threshold | Scattering CS (nm^2)
All files used herein	Samples and Calibrators	Violet SSC	SSC_1-H	2000	200	65.5	89.6	103.6	114.9	137.7	98.2	8.2

References

Welsh, J.A., Horak, P., Wilkinson, J.S., Ford, V.J., Jones, J.C., Smith, D., Holloway, J.A. and Englyst, N.A. (2019), FCMPASS Software Aids Extracellular Vesicle Light Scatter Standardization. Cytometry. doi:10.1002/cyto.a.23782

Welsh, J.A., Jones, J.C. and Tang, V.A. (2020), Fluorescence and Light Scatter Calibration Allow Comparisons of Small Particle Data in Standard Units across Different Flow Cytometry Platforms and Detector Settings. Cytometry. doi:10.1002/cyto.a.24029

Welsh, J. A., & Jones, J. C. (2020). Small particle fluorescence and light scatter calibration using FCMPASS software. Current Protocols in Cytometry, 94, e79. doi: 10.1002/cpcy.79

B: Exported FCM_{PASS} plots for light scatter and fluorescence calibration.

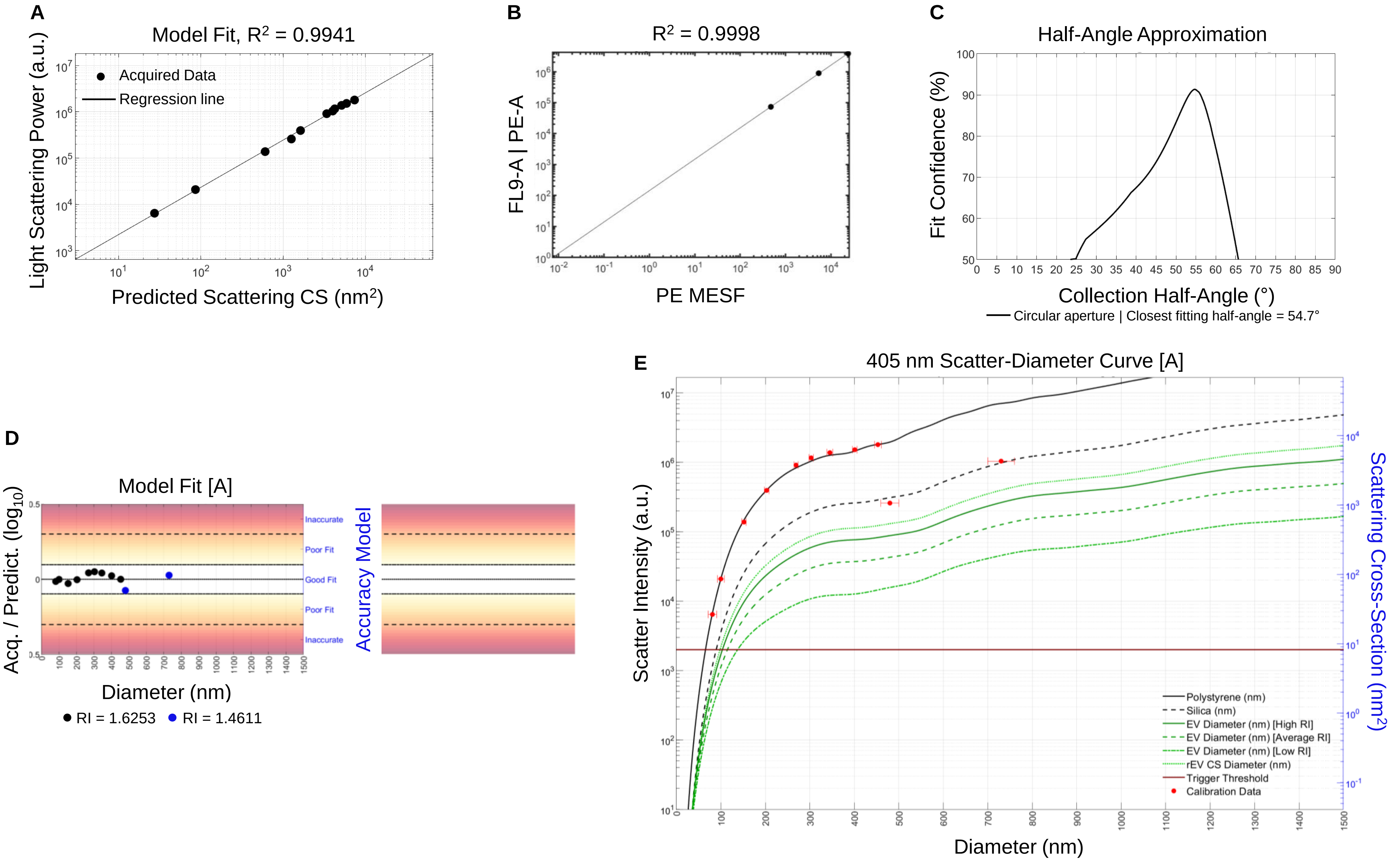

Supplement: Table S1 — FCMpass output report. [file jvi.00363-24-s0002.pdf]
