# Supplementary material for: Virion-incorporated CD14 enables HIV-1 to bind LPS and initiate TLR4 signaling in immune cells
Source: J Virol. 2024 Apr 25;98(5):e00363-24. doi: 10.1128/jvi.00363-24 (PMC11092368; doi:10.1128/jvi.00363-24)
Supplement: Table S2 — MIFlowCyt-EV checklist. [file jvi.00363-24-s0003.pdf]

Table S2. MIFlowCyt-EV checklist in compliance with the MIFlowCyt-EV framework for standardized reporting of nanoscale cytometry.

Framework Criteria	What to report	Completed criterion
1.1 Preanalytical variables conforming to MISEV guidelines.	Preanalytical variables relating to EV sample including source, collection, isolation, storage, and any others relevant and available in the performed study.	Pseudovirus used in FVM were produced as described in Materials and Methods under Section 2.4, 'Pseudovirus Production.' No additional isolation or purification techniques were used.
1.2 Experimental design according to MIFlowCyt guidelines.	EV-FC manuscripts should provide a brief description of the experimental aim, keywords, and variables for the performed FC experiment(s) using MIFlowCyt checklist criteria: 1.1, 1.2, and 1.3, respectively. Template found at www.evflowcytometry.org.	
2.1 Sample staining details	State any steps relating to the staining of samples. Along with the method used for staining, provide relevant reagent descriptions as listed in MIFlowCyt guidelines (Section 2.4 Fluorescence Reagent(s) Descriptions).	Virus samples were stained using PE-conjugated anti-CD14, Alexa Fluor 488-conjugated LPS, or biotinylated LPS followed by PE-conjugated streptavidin. Staining protocols are outlined in Materials and Methods Section 2.5, and optimizations are described in Results Sections 4.2 and 4.3.
2.2 Sample washing details	State any steps relating to the washing of samples.	No washing was performed
2.3 Sample dilution details	All methods and steps relating to sample dilution.	Labeled virus samples were routinely diluted 1:500 or 1:1000 in PBS before acquisition.
3.1 Buffer alone controls.	State whether a buffer-only control was analyzed at the same settings and during the same experiment as the samples of interest. If utilized it is recommended that all samples be recorded for a consistent set period of time e.g. 5 minutes, rather than stopping analysis at a set recorded event count e.g. 100,000 events. This allows comparisons of total particle counts between controls and samples.	Buffer only controls (PBS) were routinely included with each acquisition under the same parameters.
3.2 Buffer with reagent controls.	State whether a buffer with reagent control was analyzed at the same settings, same concentrations, and during the same experiment as the samples of interest. If used state what the results were.	Controls comprising media only with antibodies or LPS were not used in this study. Instead, we relied on virus samples devoid of the specific antigen targeted by antibody staining, which consisted of the same media composition.
3.3 Unstained controls.	State whether unstained control samples were analyzed at the same settings and during the same experiment as stained samples. If used, state what the results were, preferably in standard units.	Unstained virus samples were run at the same settings and can be found within the following figures with standard units (MESF and scattering cross section): Figs. S1C, S2C, 3A, 3C.
3.4 Isotype controls.	The use of isotype controls is applicable to immunofluorescence labelling only. State whether isotype controls were analyzed at the same settings and during the same experiment as stained samples. If utilized, state which antibody they are matched to, the concentration used, and what the results were (Section 4.2, 4.3, 4.4). Due to conjugation differences between manufacturers if should be stated if the isotype controls are from the same manufacturer as the matched antibodies.	A PE-conjugated isotype control antibody was used wherever anti-CD14 staining was done and analyzed at the same settings. The isotype control antibody was from the same manufacturer (BD Biosciences).
3.5 Single-stained controls.	State whether single-stained controls were included. If used state whether the single-stained controls were recorded using the same settings, dilutions, and during the same experiment as stained samples and state what the results were, preferably in standard units (Section 4.2, 4.3, 4.4).	All stains were single stains.

3.6 Procedural controls.	State whether procedural controls were included. If used, state the procedure and if the procedural controls were acquired at the same settings and during the same experiment as stained samples.	Negative control virus (i.e., devoid of specific antigen targeted with immunolabeling) were included, prepared and analyzed the same as experimental samples.
3.7 Serial dilutions.	State whether serial dilutions were performed on samples and note the dilution range and manner of testing. The fluorescence and/or scatter signal intensity would ideally be reported in standard units (see Section 4.3, 4.4) but arbitrary units can also be used. This data is best reported by plotting the recorded number events/concentration over a set period of time at different sample dilution. The median fluorescence intensity at each of the dilutions should also ideally be plotted on the same or a separate plot.	Serial dilutions were not performed on the samples specifically included in the manuscript.
3.8. Detergent treated EV-samples	State whether samples were detergent treated to assess lability. If utilized, state what detergent was used, the end concentration of the detergent, and what the results were of the lysis.	N/A
4.1 Trigger Channel(s) and Threshold(s).	The trigger channel(s) and threshold(s) used for event detection. Preferably, the fluorescence calibration (Section 4.3) and/or scatter calibration (Section 4.4) should be used in order to report the trigger channel(s) and threshold(s) in standardized units.	Trigger Channel and Threshold in calibrated and arbitrary units are summarized for each sample in the table on the 'Sample Acq Info' sheet (S1 Table).
4.2 Flow Rate / Volumetric quantification.	State if the flow rate was quantified/validated and if so, report the result and how they were obtained.	Flow Rate Volumetric quantification was performed using the application in the Beckman Coulter CytExpert acquisition software.
4.3 Fluorescence Calibration.	State whether fluorescence calibration was implemented, and if so, report the materials and methods used, catalogue numbers, lot numbers, and supplied reference units for the standards. Fluorescence parameters may be reported in standardized units of MESF, ERF, or ABC beads. The type of regression used, and the resulting scatter plot of arbitrary data vs standard data for the reference particles should be supplied.	Fluorescence calibration was performed on 1 parameter using FCMPASS software as previously demonstrated [1,2,3]. Details on the calibration reagents and regression can be found in S1 Table
4.4 Light Scatter Calibration.	State whether and how light scatter calibration was implemented. Light scatter parameters may be reported in standardized units of nm2, along with information required to reproduce the model.	Light scatter calibration was performed using FCMPASS software as previously demonstrated [1,2,3]. Details on the reagents and modelling parameters can be found in S1 Table
5.1 EV diameter/surface area/volume approximation.	State whether and how EV diameter, surface area, and/or volume has been calculated using FC measurements.	Diameter approximation using light scattering was not reported in the current manuscript. Units of light scatter were reported as scattering cross section (nm^2).
5.2 EV refractive index approximation.	State whether the EV refractive index has been approximated and how this was done.	N/A - these are not EVs. Refractive index approximation was not done.
5.3 EV epitope number approximation.	State whether EV epitope number has been approximated, and if so, how it was approximated.	Fluorescence data was reported in PE MESF with an approximated equivalence of 1:1, epitope: PE MESF.
6.1 Completion of MIFlowCyt checklist.	Complete MIFlowCyt checklist criteria 1 to 4 using the MIFlowCyt guidelines. Template found at www.evflowcytometry.org.	Completion of MIFlowCyt checklist included

6.2 Calibrated channel detection range	If fluorescence or scatter calibration has been carried out, authors should state whether the upper and lower limits of a calibrated detection channel were calculated in standardized units. This can be done by converting the arbitrary unit scale to a calibrated scaled, as discussed in Section 4.3 and 4.4, and providing the highest unit on this scale and the lowest detectable unit above the unstained population. The lowest unit at which a population is deemed 'positive' can be determined a variety of ways, including reporting the 99th percentile measurement unit of the unstained population for fluorescence. The chosen method for determining at what unit an event was deemed positive should be clearly outlined.	Refer to data presented in FCMPASS Plots (S1 Table).
6.3 EV number/concentration.	State whether EV number/concentration has been reported. If calculated, it is preferable to report EV number/concentration in a standardized manner, stating the number/concentration between a set detection range.	Virus stock concentrations are reported in the relevant figure legends in units of ng/mL of HIV-1 p24 (Gag_{p24}), a virus structural protein used routintely to estimate virus titer.
6.4 EV brightness.	When applicable, state the method by which the brightness of EVs is reported in standardized units of scatter and/or fluorescence.	Brightness was reported in PE MESF for fluoresence from antibody and LPS staining of virus samples. Light scatter was reported in scattering cross section
7.1. Sharing of data to a public repository.	Provide a link to the experimental data in a public data repository.	Data can be found on flowrepository.org under #FR-FCM-Z76D.

References

Welsh JA, Van Der Pol E, Arkesteijn GJA, Bremer M, Brisson A, Coumans F, et al. MIFlowCyt-EV: a framework for standardized reporting of extracellular vesicle flow cytometry experiments. Journal of Extracellular Vesicles. 2020;9(1):1713526.
